# Adsorption and Absorption Energies of Hydrogen with Palladium

**DOI:** 10.1021/acs.jpcc.2c04567

**Published:** 2022-08-19

**Authors:** Michael Schwarzer, Nils Hertl, Florian Nitz, Dmitriy Borodin, Jan Fingerhut, Theofanis N. Kitsopoulos, Alec M. Wodtke

**Affiliations:** † Institute for Physical Chemistry, Georg-August University Goettingen, Tammannstraße 6, Goettingen 37077, Germany; ‡ Department of Dynamics at Surfaces, Max Planck Institute for Multidisciplinary Sciences, Am Fassberg 11, Goettingen 37077, Germany; § Department of Chemistry, University of Crete, Heraklion 71003, Greece; ∥ Institute of Electronic Structure and Laser − FORTH, Heraklion 71110, Greece; ⊥ International Center for Advanced Studies of Energy Conversion, Georg-August University Goettingen, Tammannstraße 6, Goettingen 37077, Germany

## Abstract

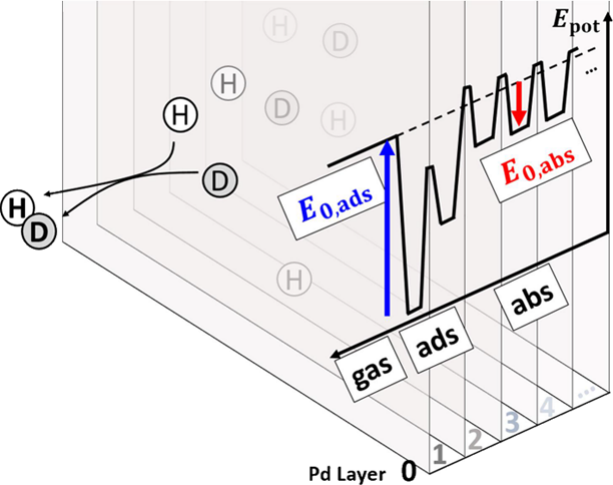

Thermal recombinative desorption rates of HD on Pd(111) and Pd(332) are reported from transient kinetic experiments performed between 523 and 1023 K. A detailed kinetic model accurately describes the competition between recombination of surface-adsorbed hydrogen and deuterium atoms and their diffusion into the bulk. By fitting the model to observed rates, we derive the dissociative adsorption energies (*E*_0, ads_^H_2_^ = 0.98 eV; *E*_0, ads_^D_2_^ = 1.00 eV; *E*_0, ads_^HD^ = 0.99 eV) as well as the classical dissociative binding energy ϵ_ads_ = 1.02 ± 0.03 eV, which provides a benchmark for electronic structure theory. In a similar way, we obtain the classical energy required to move an H or D atom from the surface to the bulk (ϵ_sb_ = 0.46 ± 0.01 eV) and the isotope specific energies, *E*_0, sb_^H^ = 0.41 eV and *E*_0, sb_^D^ = 0.43 eV. Detailed insights into the process of transient bulk diffusion are obtained from kinetic Monte Carlo simulations.

## 1 Introduction

The dissociation of molecular hydrogen on palladium surfaces leads efficiently to dissolution of the atoms into the bulk and represents arguably the most important prototype for fundamental studies of hydrogen incorporation into metals.^1−3^ It is also of practical importance; palladium is used in hydrogen fuel cells, for hydrogen storage and purification, and for hydrogenation catalysis.^4−8^ A clear and quantitative understanding requires knowledge of the kinetics of dissociative adsorption, recombinative desorption, and H atom diffusion into the bulk. This approach was demonstrated in the seminal 1979 work of Engel and Kuipers, who measured thermal rates of recombining H and D using molecular beam relaxation spectrometry (MBRS).^3^ Their data analysis required several assumptions including the following: neglecting the H/D isotope effect, approximating the 2nd order recombination by an effective 1st order reaction, and using a perturbation approximation to model diffusion into the bulk. Furthermore, strictly speaking rate constants were not directly determined in that work. Instead, the adsorption enthalpy reported by Conrad et al.^1^ was used as an activation energy for adsorption, and Arrhenius prefactors were varied to fit the temperature-dependent rate data. It is worth noting that the work of Conrad et al.^1^ remains the only report of an experimentally derived dissociative adsorption enthalpy of hydrogen on Pd(111).

The intervening four decades have seen remarkable experimental as well as computational advances in our ability to investigate surface reaction kinetics. Velocity-resolved kinetics (VRK) is a recently developed variant of MBRS that initiates a surface reaction with a temporally narrow pulsed molecular beam and subsequently provides the flux of desorbing products as a function of reaction time at the surface, also known as the kinetic trace.^9−19^ VRK can provide surface site-specific reaction rate constants^17^ as well as surface diffusion rate constants.^10,11^ Computational methods have also improved dramatically. For example, density functional theory (DFT) has become a workhorse for computing fundamental adsorbate–surface interactions,^20,21^ which can be helpful in modeling thermal rate coefficients, providing key parameters needed for implementing transition state theory.^22^ Advances in computer hardware and software also allow for numerical modeling of complex kinetic mechanisms, for example, using kinetic Monte Carlo methods.^23^ This overcomes the sometimes severe approximations about mechanisms that have been required in the past when using analytic expressions to fit kinetic data. In light of these methodological improvements and the importance of the hydrogen palladium system, we decided it worthwhile to revisit this problem.

Specifically, we have obtained recombination and diffusion rate constants from newly acquired VRK data for surface temperatures between 523 and 1023 K. Using a numerical grid-hopping model of recombination and diffusion to fit the VRK data allowed us to derive the dissociative adsorption energy of H_2_ and its isotopologues to Pd as well as accurate values for H and D absorption energies. We also implement a tracer kinetic Monte Carlo method, which allows visualization of the dissolution of hydrogen into palladium.

## 2 Methods

### 2.1 Experimental Setup

The ultrahigh vacuum apparatus (base pressure <2 × 10^–10^ mbar) used in this study has been described elsewhere.^15,17^ Prior to kinetic trace measurements, the surface is cleaned with Ar-ion sputtering (15 min, 3 kV) and annealed (1020 K for 15 min, followed by 1 min at 1170 K). Surface cleanliness is verified by Auger electron spectroscopy. A palladium single crystal (1 cm diameter) with two facets is used (MaTeck GmbH). The upper half of the circular face of the crystal has a (111) orientation, and the lower half has a (332) orientation. The incident molecular beam is smaller than 2 mm (FWHM), see SI Section 1, and only illuminates the desired surface, ensuring that reactive signal is specific to the individual facet. The surface facet of interest was positioned in front of the detector such that the molecular beam hits its center. The alignment was verified using a laser diode. The step density of the (111) crystal is estimated from the uncertainty of the cut angle to be 0.1–0.2%. The temperature of the surface sample can be varied from room temperature up to the melting point by electron bombardment heating and was measured by a Chromel/Alumel thermocouple junction placed inside the crystal. The temperature is regulated using a proportional-integral-differential device capable of varying the current of the electron-emission filament.

For kinetic measurements, a homebuilt piezo-actuated pulsed valve produced molecular beam pulses of H_2_/D_2_ mixtures with 35 μs FWHM duration. The absolute molecular flux, which is required for the analysis of experimental data, was obtained using a calibration procedure presented in SI Section 1. The molecular beam was incident at 30° from the surface normal, allowing product detection near the surface normal without disturbance from the scattered incident beam, e.g., due to space-charge effects. The kinetic energy component perpendicular to the surface of a mixed H_2_/D_2_ beam was determined to be 0.06 eV for H_2_ and 0.12 eV for D_2_.

Recombination products were ionized 20 mm from the surface by nonresonant multiphoton ionization using a Ti:Sapphire laser (35 fs, 0.5 W at 1 kHz) producing ions that were extracted perpendicular to the imaging plane by a homogeneous electric field formed with a grounded extractor grid (1000 LPI, Cu) and a repeller electrode. The ions were mapped onto a flight-time gated stack of two microchannel plates (MCP) in Chevron configuration. This allowed for detection of HD and discrimination against H_2_ and D_2_. The image of a phosphor screen placed behind the MCP was recorded with a CCD camera. Ion images were recorded for many delay times between the pulsed molecular beam and the ionizing laser. This detector geometry records the in-plane scattering events; hence, the velocities of both reactant and product molecules can be obtained from the position in the image. The velocity information is used for two important transformations. First, the flight time of the products from the surface to the ionizing laser can be calculated, allowing the reaction time at the surface to be found. Second, the product flux is obtained, which is proportional to the rate of the reaction. The product flux as a function of reaction time is referred to as the kinetic trace. Kinetic traces were obtained at surface temperatures between 523 and 1023 K using beams with three H_2_/D_2_ mixing ratios 1:1, 3:1, and 9:1.

### 2.2 Kinetic Model

We use a numerical grid-hopping formalism to model the kinetics of recombinative desorption and diffusion into the bulk. In this formalism, subscripts are used to indicate positions in the grid: e.g., 0 refers to the surface, 1 to the first subsurface site, and so on. Equations 1, 2, 3, and 4 show the rate equations that control population in the first two grid points of the model.
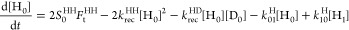
1
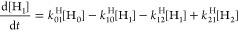
2
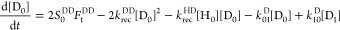
3
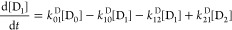
4Here, time-dependent concentrations at each grid point *i* = 0,1, ... are indicated by brackets [H_*i*_] or [D_*i*_]. The reaction is initiated by a time-dependent dose of H (2*S*_0_^HH^*F*_t_^HH^) or D atoms (2*S*_0_^DD^*F*_t_^DD^), given by the transient molecular beam flux *F*_t_^HH^ or *F*_t_^DD^ (see SI Section 1) and the sticking probability *S*_0_^HH^or *S*_0_^DD^ at the experimental incidence energies of the molecular beam. Rate constants for recombinative desorption are denoted as *k*_rec_^HH^, *k*_rec_^HD^, and *k*_rec_^DD^, and site hopping rate constants moving the concentration from grid point *i* to grid point *j* are denoted as *k*_*ij*_^H or D^. For [H_*i* > 7_] and [D_*i* > 7_], coarse graining is necessary—we accomplished this with an exponentially growing grid size (see SI Section 2).

Propagating these equations numerically leads to the time-dependent surface concentrations of both isotopes [H_0_] and [D_0_]. In the experiment, the molecular beam impinges periodically at a rate of 25 Hz onto the surface. Due to the absorption of H into the deep bulk, it is not expected that the system releases all H and D atoms before the next pulse arrives. As a consequence, after an induction period where the surface takes up more H and D atoms than it releases via recombination, a steady-state condition is established, meaning that the transient HD formation is the same for each pump pulse. The transient HD formation rate is computed from eq 5 and compared to VRK experimental rates.
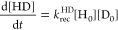
5

**Figure 1:**
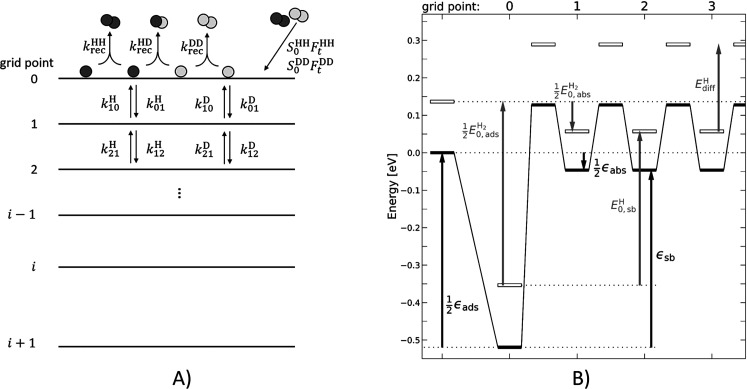
(A) Schematic diagram showing the kinetic processes of dissociative adsorption of molecular hydrogen and diffusion to the bulk of the resulting atoms. (B) Schematic diagram of the reaction coordinate. Solid black lines indicate the classical energy diagram. The zero-point energy corrected energies are also shown for H. Relevant energies introduced in the text are also shown. The energy scale in the *y* axis refers to a single atom. The 1st subsurface site is not isoenergetic with the bulk sites.^24^ Since the analysis of this work is insensitive to this fact, we have represented the reaction coordinate in a simplified form.

To fit VRK data, we must now optimize recombination *k*_rec_^AB^(*T*) and site hopping *k*_*ij*_^A^(*T*) rate constants for two isotopes—here, A and B can be either H or D as shown. This problem is illustrated in Figure 1.

Due to the large number of rate constants involved in the grid hopping of an isotopic mixture, we must simplify the problem. We first assume that all diffusional site-hopping rate constants are equal to those for site hopping in the Pd bulk. Only the endoergic hopping from the surface to the 1st subsurface site *k*_01_^A^(*T*) is considered distinct.
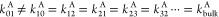
6

This simplification does not affect the outcome of the kinetic model compared to a model with individual *k*_*ij*_^A^(*T*), as under our experimental conditions *k*_*ij*_^A^(*T*) are much faster than recombination. A fast equilibration between the surface and the near bulk is established, and the magnitude of the first few *k*_*ij*_^A^(*T*) is not sensitive to the simulation. *k*_bulk_^A^(*T*) is obtained from previously reported bulk diffusion measurements.^25−27^ Diffusion constants are fitted accurately with an Arrhenius expression to characterize their temperature dependence and are converted to site-to-site hopping rate constants (see SI Section 3). Having assumed that *k*_10_^A^(*T*) = *k*_bulk_^A^, we may find *k*_01_^A^ in terms of an equilibrium constant that can be expressed in the language of statistical mechanics as follows:
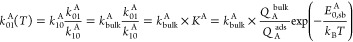
7Here, *E*_0, sb_^A^ is the zero-point energy corrected energy required to move a surface-bound H or D atom to the bulk and *Q*_A_^bulk^ is the partition function for that atom bound in the bulk. *Q*_A_^bulk^ was computed using a 1D model potential describing the interaction of H or D in Pd bulk, parameters of which were derived by fitting to the present kinetic data as well as previously published absorption enthalpy data.^28^ See SI Sections 4 and 5 for more details.

We also employ a model of isotope specific recombination rate constants *k*_rec_^AB^(*T*) described by eq 8, which has been shown to be highly accurate for H and D recombination on Pt.^29^
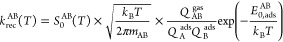
8Here, *S*_0_^AB^(*T*) is the thermal sticking coefficient obtained by averaging known energy-dependent sticking probabilities^30,31^ over a Maxwell Boltzmann distribution at temperature *T*, see SI Section 6. *Q*_AB_^gas^ is the partition function for the gas-phase molecule and is well known, eq S26 of the SI. *Q*_A_^ads^ is the adsorbate partition function and is computed as previously reported, where electronic-spin is properly accounted for and adsorbate wavefunctions and eigen energies are computed within the static surface approximation using a DFT-based potential energy surface (PES).^29^*E*_0, ads_^AB^ is the dissociative adsorption energy of isotopologue AB. See SI Section 7 for more details.

Five isotope independent model parameters were varied in the fitting process: the isotope independent adsorption energy ϵ_ads_ and absorption energy ϵ_abs_, as well as three bulk model potential parameters (*w*, *f*, and *g*) that determine *Q*_A_^bulk^ (see SI Section 4). We optimize these parameters to produce a best fit to the rates of HD formation obtained in our VRK experiments on Pd(111) and Pd(332) between 523 and 1023 K with three H_2_/D_2_ mixing ratios. Since our DFT calculations indicate no preference for hydrogen atoms at steps, we use the same adsorption energy for Pd(111) and Pd(332). To reduce the correlation error in the fitting, we simultaneously fit the VRK data obtained in this work as well as previously reported temperature-dependent absorption enthalpy data.^32^

An uncertainty estimation of the model parameters was carried out based on the two quantities, which are thought to be most error prone. (1) The flux of H atoms to the surface due to the molecular beam. The uncertainty of the procedure presented in SI Section 1 was estimated to be ±30%. (2) The DFT-based PES used for the partition function of H interaction with the Pd surface. The uncertainty was evaluated by using besides the present PBE PES also a RPBE PES from ref (33) to calculate the partition function of H adsorbed on Pd. Usually, PBE and RPBE span an upper and a lower border of feasible interaction energies, respectively.^34,35^ This is also observed for dissociative adsorption of H_2_ on Pd (see Section 4.2). See SI Section 8 for the result of the uncertainty estimation.

## 3 Results

**Figure 2:**
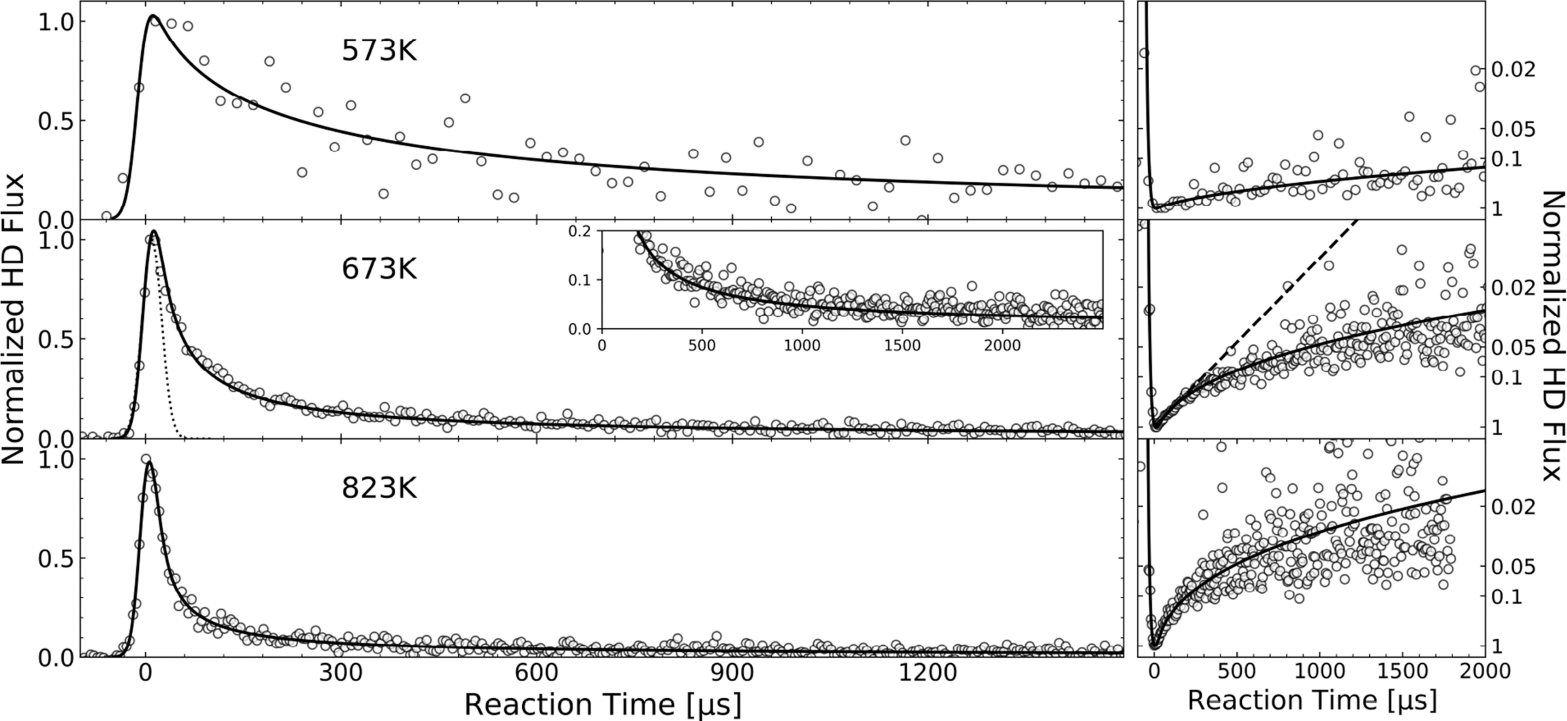
HD formation rate on Pd(111) as a function of reaction time (open circles). Solid lines show the best fit of the kinetic model involving recombinative desorption and diffusion into the bulk. The inset for the 673 K data shows that the long-time behavior is reproduced well by the model. The right column panels show the data after attempted linearization using eq 11. The dashed line shows the behavior expected from a 2nd order reaction ignoring bulk diffusion. The time dependence of the dosing pulse is shown as a dotted line. H_2_ and D_2_ were dosed with 1.35 and 1.95 × 10^–3^ ML per pulse, respectively.

Figure 2 (left column) displays three examples of experimentally derived kinetic traces—HD production rates *r*_HD_( ≡ d[HD]/d*t*) as a function of reaction time—obtained with VRK using Pd(111). Kinetic traces obtained using Pd(332) reflect slightly faster rates but are nearly indistinguishable from those obtained with Pd(111)—SI Section 9. Insights into the processes giving rise to the form of the kinetic traces can be found by assuming a 2nd order recombination of two identical atoms (eq 9).
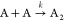
9

In this case, the rate of A_2_ formation is given by eq 10:
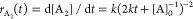
10Here, *k* is the 2nd order recombination rate constant and [A]_0_ is the initial concentration of A. A simple linearization of the peak-normalized data is predicted from eq 11.
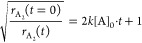
11

This is shown in one panel of the right column of Figure 2 as a dashed line. The observed time-dependent rates deviate strongly; furthermore, neither a reasonable isotope effect nor the effect of spatial inhomogeneity of [H]_0_ and [D]_0_ produced by molecular beam dosing can explain this deviation.

Hydrogen diffusion constants in bulk Pd have been previously reported,^25−27,36^ from which we can estimate typical diffusion lengths under our experimental conditions—we find that H may easily diffuse 1–10 μm through the bulk in the 1 ms time scale typical of our experiments. Furthermore, less energy is required for adsorbed H atoms to be absorbed into the bulk of Pd than is required for recombinative desorption—see Figure 1B.^32^ These insights strongly suggest that absorption and bulk diffusion of H and D can compete with recombinative desorption under the conditions of our experiments.

**Figure 3:**
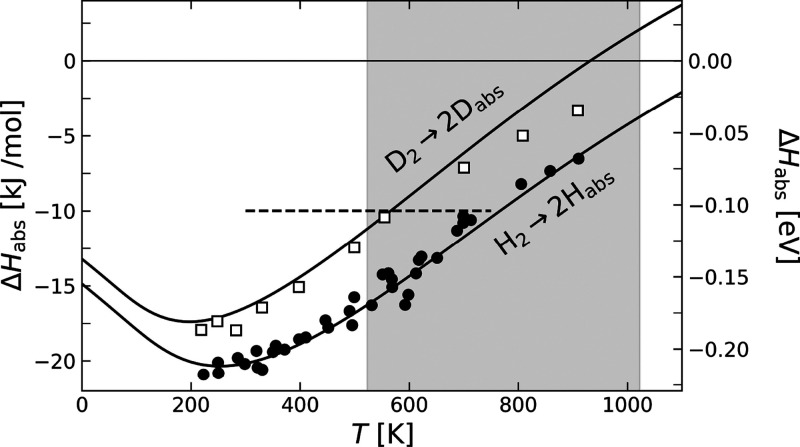
Temperature dependence of the absorption enthalpy Δ*H*_abs_ for hydrogen and deuterium: H_2_ → 2H_bulk_ (filled circles)^32^ and D_2_ → 2D_bulk_ (open squares).^37^ The solid line shows the fit obtained using the desorption/diffusion model described in the Methods section. The fit relies on three parameters describing the 1D diffusional potential of H_bulk_/D_bulk_, from which the partition function of H_bulk_/D_bulk_ was computed. The dashed line marks the temperature and isotope independent value obtained by Engel and Kuipers.^3^

The solid lines in Figure 2 show the predictions of our kinetic model of recombinative desorption and diffusion—the resulting simultaneously fitted temperature-dependent absorption enthalpy data of ref (32) are shown in Figure 3. The quality of the simultaneous fits to both data sets is excellent. The ability of the desorption/diffusion model to fit both the VRK data from the (111) and (332) surface as well as the enthalpy data confirms that diffusion into the bulk is primarily responsible for the deviation from the expectations for a 2nd order reaction.

It also provides strong evidence that the desorption/diffusion model reflects the fundamental properties of the system, represented by the derived fitting parameters. Table 1 provides the fundamental quantities derived from the fitting of the data in Figures 2 and 3.

**Table 1:** Recommended Adsorption and Absorption Energies for Hydrogen Interacting with Palladium^a^

*E*_0, ads_^AB^^b^	H_2_ → 2H*	HD → H* + D*	D_2_ → 2D*
	0.98	0.99	1.00
*E*_0, abs_^AB^^c^	H_2_ → 2H_(abs)_	HD → H_(abs)_ + D_(abs)_	D_2_ → 2D_(abs)_
	–0.159^e^	–0.153	–0.140^f^
*E*_0, sb_^A^^d^	H* → H_(abs)_		D* → D_(abs)_
	0.41		0.43

a See Figure 1B for parameter definitions. All values are in eV.

b The isotope independent classical energy of dissociative adsorption is ϵ_ads_ = 1.02 ± 0.03 eV.

c The isotope independent classical energy of the gas phase to bulk absorption is *ϵ*_abs_ = – 0.093 ± 0.005 eV.

d The classical energy required to move an H or D atom from the surface to the bulk is *ϵ*_sb_ = 0.46 ± 0.01 eV.

e Combined plane wave and localized basis set method (CPLB) from ref (38) predicts −0.292 eV. See the Discussion Section.

f CPLB from ref (38) predicts −0.184 eV. See the Discussion Section.

We also provide the isotope specific rate constants for recombination and subsurface site hopping as a function of temperature in SI Section 10 using a simple parametrization, as the use of eqs 7 and 8 can be somewhat tedious.

## 4 Discussion

In this work, we have developed a model for the hydrogen palladium system that consistently and quantitatively reproduces VRK, sticking probability and absorption enthalpy measurements for two isotopes and for two surface facets. The fundamental quantities derived from this model are both energetic (dissociative adsorption and absorption energies) and entropic (partition functions for adsorbed and absorbed atoms). This allows us to predict rate constants for recombinative desorption and bulk penetration over a wide range of conditions and to model the kinetic pathways that lead gas-phase hydrogen to become dissolved as atoms in bulk Pd. In the following section, we discuss the consistency of the recombination rate constants predicted by our model with previously reported data, much of which comes from experiments carried out at much lower temperatures. We also compare our experimentally derived energies to results from electronic structure theory. In the final section of this discussion, we show examples of how the validated desorption/diffusion model can be used to explore the diffusion properties of this important system.

### 4.1 Comparison to Prior Kinetic Results for Hydrogen Recombination

**Figure 4:**
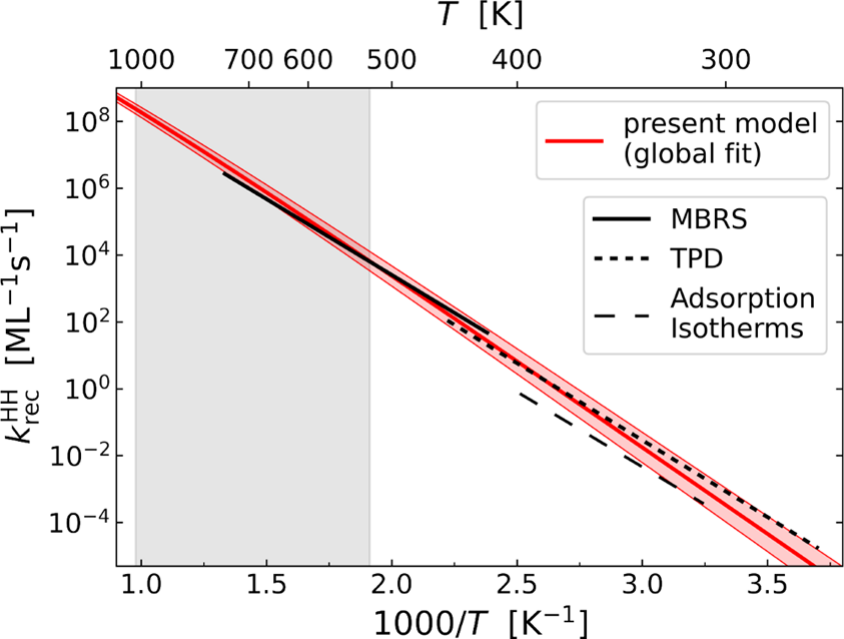
Comparison of the H + H recombination rate constant on Pd(111) obtained in this work with previous reports. The rate constants obtained from the fundamental quantities of Table 1 are shown as a red line with a shaded uncertainty range. The gray shaded region represents the temperature range studied in this work. The results of MBRS rate constants (ref (3)) are shown as a black solid line, and those obtained with TPD from ref (2) are shown as a black dotted line (see SI Section 12). Rate constants obtained from adsorption isotherms in ref (1) are shown as a black dashed line (see SI Section 11).

Figure 4 shows the desorption/diffusion model’s fitted results for recombinative desorption rate constants for H + H on Pd(111) from 300 to 1000 K (red line with an uncertainty band). The MBRS results of ref (3) (solid black line) can be compared directly. Generally, they are in good agreement; however, their temperature dependence appears weaker than that of the recombination rate constants found in this work. As mentioned in the introduction, the MBRS study did not derive rate constants directly. Instead, they used a *T*-independent activation energy equal to the 0.9 eV adsorption enthalpy reported by Conrad et al.^1^ obtained between 300 and 400 K and fit their kinetic data by varying the rate constant’s pre-exponential factor, assuming an Arrhenius form. Hence, the temperature dependence of their data analysis model was constrained. The diffusion/desorption model is not constrained in this way, a fact that suggests the difference in temperature dependence seen between red and the black curves in Figure 4 is real. This could be explained if the adsorption enthalpy reported by Conrad et al. required revision upward. Also the temperature and isotope independent H_2_ absorption enthalpy obtained by Engel and Kuipers shows a systematic deviation with respect to literature (see Figure 3). This small inaccuracy could be compensated with an upward correction of the adsorption enthalpy, while keeping the surface to bulk energy difference fixed.

The adsorption enthalpy, reported by Conrad et al., was derived from Langmuir isotherm data. In SI Section 11, we show how to derive rate constants for recombinative desorption from their data with the help of thermal sticking probabilities, which we consider highly reliable. Briefly, the recombination rate constants are modeled with an Arrhenius form whose activation energy is weakly coverage dependent and the equilibrium coverage from the isotherm data is obtained from a calculation of the kinetic steady state. Figure S7 shows the excellent fit to the isotherm data obtained in this way. The recombination rate constants that reproduce the isotherm data are shown in Figure 4 as a black dashed line. At the lowest temperatures, they are within the uncertainty bands of our model predictions but deviate significantly at higher temperatures. The temperature dependence of the rate constants is again too weak; in fact, these rate constants are inconsistent with the higher temperature MBRS-derived rate constants, even though they rely on the same adsorption data. This indicates a possible error in the isotherm experiments of Conrad et al. In that work, it was assumed that changes in the observed work function were linearly dependent on the coverage. Should this assumption fail, temperature-dependent deviations might arise as seen in Figure 4.

Additional low-temperature data support this statement. Assuming an Arrhenius form whose activation energy is coverage dependent, we obtained an excellent fit to TPD data of ref (2)—see SI Section 12. The derived recombinative desorption rate constants are also shown in Figure 4 as a black dotted line. These rate constants are significantly larger than those obtained from the isotherm experiments, and they are in good agreement with the predictions of the desorption/diffusion model. They are also consistent with rate constants obtained from MBRS when extrapolated to higher temperatures. We note that the VRK, MBRS, and TPD derived rate constants all fall within the uncertainty bands of the predictions of our desorption/diffusion model. This discussion strongly suggests that the results relying on the adsorption enthalpy reported by Conrad et al. are inconsistent with the other data available and supports statements above suggesting a problem with the isotherm measurements. This leads us to conclude that the energetic parameters derived from the desorption/diffusion model (Table 1) are currently the most accurate available.

### 4.2 Benchmarks for Electronic Structure Theory

We next compare the energies of adsorption and absorption derived from this work with DFT calculations performed by us as part of this work and with published results. This comparison is best accomplished using isotope independent classical energies shown schematically in Figure 1 and presented in the footnotes of Table 1. Table 2 shows these comparisons; we restrict our comparison to the most frequently used GGA functionals—PW91, PBE, RPBE, and BEEF-vdW.

**Table 2:** Comparison of Derived Adsorption (ϵ_ads_), Absorption (ϵ_abs_), and Surface to Bulk (ϵ_sb_) Energies to Various DFT Studies^a^

	method	ϵ_ads_^e^	ϵ_abs_	ϵ_sb_
experiment	this work des/dif. Model	1.02 ± 0.03	–0.093 ± 0.005	0.46 ± 0.01
theory	RPBE	0.79,^39^	**0.11**^b^	0.45,^39^^d^
		0.84,^33^		0.475,^33^^d^
		0.91^40^		0.501,^40^^d^
	PW91	1.18,^24^	–0.28^24^^c^	0.45^24^
		1.18,^41^		
		1.20^40^		
	PBE	**1.14**,^b^	**–0.16**,^b^	**0.49**,^b^
		1.26,^42^	–0.58^42^	0.34^42^
		1.09,^38^	–0.10^38^	0.50^38^
		0.94^40^		
	BEEF-vdW	0.69^40^		

a All values are in eV.

b Present DFT calculations.

c Gas phase to the 2nd subsurface layer.

d Calculated assuming *ϵ_abs_* = 0.11 *eV*.

e All reported DFT calculations for ϵ_ads_ used a 2 × 2 unit cell, except for ref (38) where a 4 × 4 unit cell was used.

The adsorption (ϵ_ads_∼1.0 ± 0.2 eV) and absorption (ϵ_abs_∼ – 0.1 ± 0.2 eV) energies predicted by DFT depend strongly on the choice of functionals. Even within one choice of functional, results are not identical, highlighting other methodological differences between calculations. As is commonly reported, RPBE functionals lead to somewhat lower adsorption energies and both PW91 and PBE functionals lead to somewhat higher values. The PBE results of ref (42) seem to be outliers within this comparison group, suggesting caution in their use. The BEEF-vdW functional underestimates the adsorption energy by the greatest amount.^40^ It is worth emphasizing that computed values of ϵ_sb_, the classical energy required to move an H atom from the surface to the bulk, are quite accurate and relatively independent of DFT methodology. This points out again the well-known observation that DFT errors are smaller when comparing energies between two solid-state systems, where the absolute errors of DFT are similar and tend to cancel.

It is also interesting to compare to recent calculation that goes beyond the usual DFT approach. Here, Sakagami et al.^38^ reported results of a combined plane wave and localized basis set (CPLB) approach. CPLB energies for absorption of one H_2_ or D_2_ molecule into the bulk of Pd were reported to be *E*_0, abs_^H_2_^ = – 0.292( – 0.16) eV and *E*_0, abs_^D_2_^ = – 0.184( – 0.14) eV. Here, experimental values from this work are shown in parentheses.

### 4.3 Microscopic Insights into H Recombination on Pd

**Figure 5:**
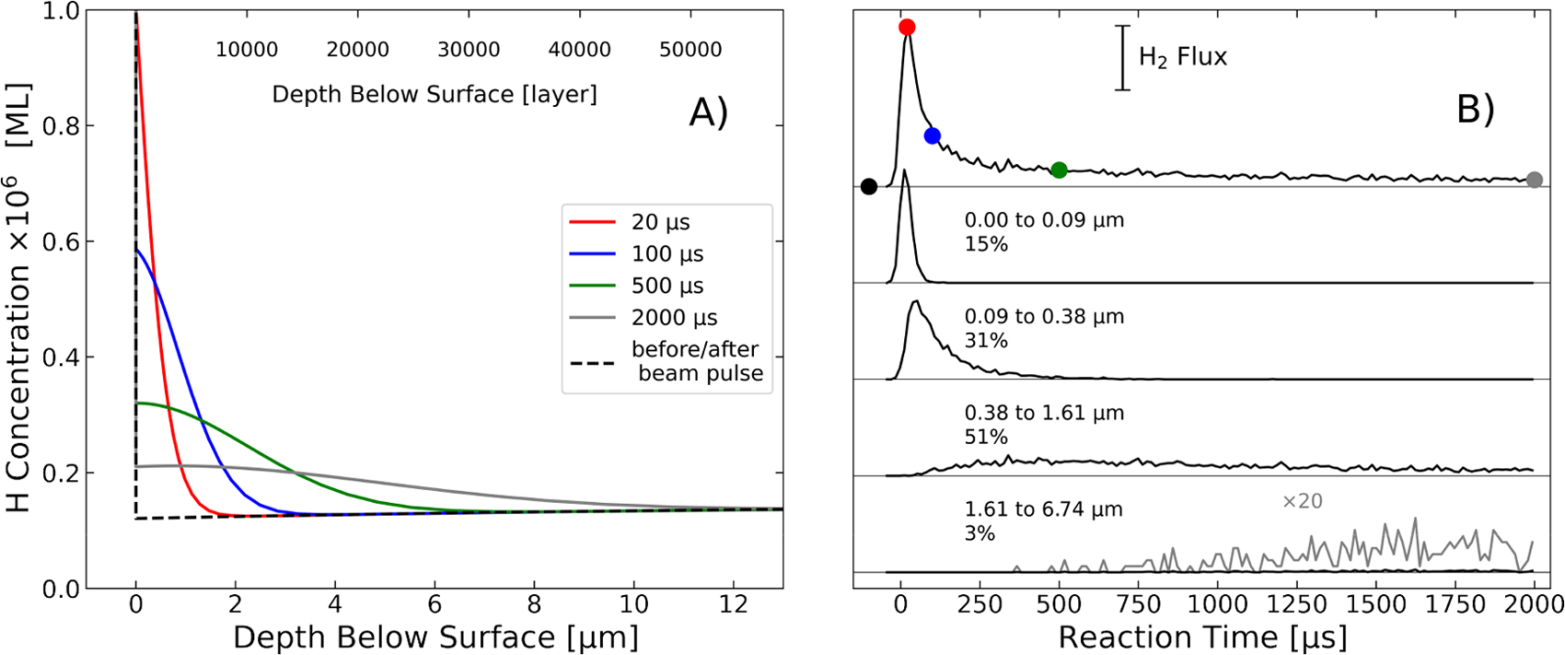
(A) Spatial distribution of H atom concentration in the bulk of Pd simulated using the mean field model (673 K, 3 × 10^–3^ ML H_2_ dose). The colored lines show the distribution at different times after the molecular beam initiated the reaction (these timings are indicated in panel B as colored points). Notice that the H concentration on the surface is higher by a factor of about 1000 at any times and not visible in the plot. (B) Results from the TkMC simulation. The upper trace corresponds to the whole flux of recombining H atoms. The traces below are partial traces showing H atoms, which reached a specific maximum penetration depth within the indicated borders. The sum of partial traces yields the total trace.

Having an experimentally validated kinetic model of desorption and diffusion allows us to explore the temporal dependence of bulk hydrogen-atom concentration profiles induced by molecular hydrogen dissociation on Pd surfaces. Figure 5A shows profiles like this under conditions typical of our experiments, obtained by solving the mean field kinetic equations, as explained in Section 2.2. Here, a pulse of H_2_ lasting ∼35 μs doses 3 × 10^–3^ ML of H_2_ onto a Pd(111) surface at 400 °C. Already during the dosing time, the bulk concentrations extend more than 1 μm into the solid (red curve). At later times, the influence of bulk diffusion is clearly seen as a broadening of the H-atom concentration profiles and a drop in the surface concentration. This reduction in the surface concentration results in the slower than expected recombinative desorption rate, described in the right panel of Figure 2.

How bulk diffusion influences the kinetic trace observed in the VRK experiments can be seen in even greater detail using a Tracer Kinetic Monte Carlo (TkMC) approach, described in detail in SI Section 13. In TkMC, we follow each of kMC trajectories of individual hydrogen atoms subject to diffusion and recombination at the surface. From that information, we decompose the kinetic trace, showing at each time to what extent these detected products result from atoms that made excursions into the bulk. In TkMC, we calculate trajectories in the usual kMC fashion, using importance sampling to evaluate the probability of individual site-to-site transitions. However, the importance sampling makes use of time-dependent mean-field concentration profiles, like those shown in Figure 5A to avoid the need to simulate a large-scale multiparticle problem.

Figure 5B shows the outcome of these TkMC simulations. In each trajectory, a single hydrogen atom initially located at the surface hops to the subsurface and exhibits diffusional motion toward or away from the surface. Recombination can occur when the projectile again reaches the surface. A total number of 100,000 single-atom trajectories was produced, of which ∼10,000 lead to desorption within 2000 μs. The upper panel shows the overall kinetic trace as seen in the experiment. It matches the solutions found from the mean field equations. The panels below show the time-dependent H_2_ production resulting from specific groups of H atoms, sorted by their maximum penetration depth into the bulk. One sees a strong correlation between the maximum penetration depth and the time at which products are formed. Note that this simulation only provides information about one of the recombining atoms—the history of the partner atom is not known in detail but is rather given by the mean field solutions.

### 4.4 Comparison of Pd(332) and Pd(111) Kinetic Traces

It is a remarkable outcome of this work that the recombination kinetics are indistinguishable, when comparing reaction on Pd(111) with that on (332). This observation is supported by the present DFT calculations, which predict dissociative adsorption energies of 1.14 eV on Pd(111) and 1.12 eV on Pd(332). If DFT were wrong and there were an energetic stabilization of H on the surface steps for Pd(332), reduced rates for recombination and diffusion to the bulk would result for this facet compared to Pd (111). This would be clearly seen in the experimental data. DFT also shows that the adsorbate potential energy surfaces for the two facets are quite similar; see Figure S5. This is why the partition functions and prefactors are similar on the two facets. With similar partition functions and binding energies, the rate constants are predicted to be nearly equal as observed in the experiment. The insensitivity to steps of the hydrogen recombination rate constant on Pd contrasts behavior reported for Pt.^29^ H recombination on Pt(332) was found to be significantly faster than on Pt(111) under similar experimental conditions to the current study. This is consistent with the non-zero step stabilization energy of H on Pt and a facet-dependent partition function.^29^ We emphasize that the experiments reported in ref (29) were carried out using the same apparatus as in this work. This comparison gives us additional confidence of the insensitivity to steps observed in this work.

## 5 Conclusions

In this work, we investigated the recombinative desorption rate of hydrogen on Pd(111) and Pd(332) with VRK. Consistent with previous work by Engel and Kuipers,^3^ we find that the transient rates are strongly affected by diffusion of hydrogen atoms into the bulk. In addition to our VRK data, we simultaneously fitted previously measured absorption enthalpies with a detailed recombination-diffusion kinetic model. The model is based on the fundamentals of statistical mechanics, especially the principle of detailed balance and relies on an ab initio calculation of the entropy of the adsorbed H atom. The success of the model allows us to derive accurate adsorption and absorption energies for the isotopes of hydrogen on Pd(111) and (332).

The comparison of our hydrogen atom recombination rate constants on Pd(111) with previous reports demonstrates the fidelity of the employed model—the previously reported recombination rates are reproduced between 250 and 1050 K. We find that the recombination rate constant on Pd(332) is only slightly higher than that on Pd(111), which is fully explained by the changes of the adsorbate entropy, deduced from DFT calculations. We find no energetic preference for hydrogen atoms at B-type steps.

The fitted reaction–diffusion kinetic model was also used to provide a microscopic picture of hydrogen atom competition between recombination and subsurface diffusion on Pd(111). We implemented a Tracer Kinetic Monte Carlo method to explore the fate of hydrogen atoms prior to a recombination event. We find that hydrogen atoms, which penetrate deeper into the bulk, have an increased residence time at the crystal, which marks the strong influence of H diffusion into palladium for recombinative desorption.

## Supporting Information Available

Additional details on the molecular beam flux calibration, coarse-grained grid diffusion, the potential for H absorbed in the bulk, the thermal sticking coefficient of HD, construction of the H(D) recombination rate constant, the uncertainty estimation, kinetic data on Pd(332), a simple-to-use expression for the derived rate constants, reanalyses of TPD and isotherm data, and details on the tracer kinetic Monte Carlo method and DFT calculations (PDF)
