# Supplementary material for: Adsorption and Absorption Energies of Hydrogen with Palladium
Source: J Phys Chem C Nanomater Interfaces. 2022 Aug 19;126(34):14500–8. doi: 10.1021/acs.jpcc.2c04567 (PMC9442642; doi:10.1021/acs.jpcc.2c04567)
Supplement: Supplementary file 1 — jp2c04567_si_001.pdf [file jp2c04567_si_001.pdf]

Supporting Information for: The Adsorption and Absorption Energies of Hydrogen with Palladium

Michael Schwarzer[1], Nils Hertl[2], Florian Nitz[1], Dmitriy Borodin[1,2], Jan Fingerhut[1], Theofanis N. Kitsopoulos[1,2,3,4] *, Alec M. Wodtke[1,2,5] **

[1]Institute for Physical Chemistry, Georg-August University Goettingen, Tammannstraße 6, 37077 Goettingen, Germany.

[2]Department of Dynamics at Surfaces, Max Planck Institute for Multidisciplinary Sciences, Am Fassberg 11, 37077 Goettingen, Germany.

[3]Department of Chemistry, University of Crete, Heraklion, Greece

[4]Institute of Electronic Structure and Laser – FORTH, Heraklion, Greece

[5]International Center for Advanced Studies of Energy Conversion, Georg-August University Goettingen, Tammannstraße 6, 37077 Goettingen, Germany.

Email: *theo.kitsopoulos@mpinat.mpg.de, **alec.wodtke@mpinat.mpg.de

Contents

1. Molecular Beam Flux Calibration 2
2. Coarse Grained Grid Diffusion 6
3. Bulk Diffusion Constants for H and D 8
4. Bulk Potential and Partition Function 9
5. Modelling the Absorption Enthalpy 10
6. Thermal Sticking Coefficient 11
7. H(D)-Recombination Rate Constant 11
8. Uncertainty Range 15
9. Velocity Resolved Kinetics Data of Pd(332) 16
10. Temperature Dependent Rate Constants as Extended Arrhenius 16
11. Reanalysis of Conrad *et al.* Isotherms 17
12. Reanalysis of Gdowski *et al*. TPD Data 18
13. Tracer Kinetic Monte Carlo (TkMC) Method 19
14. Computational Details 21

References 22

1. Molecular Beam Flux Calibration

A procedure for the estimation of the number of molecules N in a molecular beam pulse was developed using laser based multi-photon-ionization and spatial ion imaging. Comparable methods have been proposed previously.[1, 2] The procedure aims to characterize the spatial molecule density D (x,y,z) produced by one pulse. The number of molecules $\mathrm{d}N$ in a volume element $\mathrm{d}V$ is given as

$$\mathrm{d}N(x,y,z) = D(x,y,z)\mathrm{d}V = D_{\max}D_x\mathrm{d}xD_y\mathrm{d}yD_z\mathrm{d}z \qquad \textit{Eq. S1}$$

In this notation $D_{\max}$ is the molecule density (molecules/ m^3) at the peak of the molecular beam density distribution. The shape of the distribution is characterized by the shape functions D_x, D_y and D_z which are assumed independent of each other. The product of three Gaussians describes the beam

shape accurately. Each shape function is peak normalized, such that the density at the center equals D_{max}.

In addition, an ionization efficiency function $I(x,y,z)$ was defined in order to account for varying molecule detection probability along the laser propagation (y-axis). The laser ionization function returns the probability of ionization at a point in space

$$I(x,y,z) = I_{\mathrm{max}} I_x I_y I_z \qquad \textit{Eq. S2}$$

where I_{max} is the probability of ionization at the center of the laser focus. In the present experiment, the laser ionization profile is narrow compared to the molecular beam pulse in x and z. Only the y component, which is the propagation axis of the laser beam, is similar in width to the molecular beam.

The number of molecules N, that are ionized by a laser pulse, is given by integration of Eq. S1 weighted by the ionization probability.

$$N = D_{\mathrm{max}} I_{\mathrm{max}} \int D_x I_x \mathrm{d}x \int D_y I_y \mathrm{d}y \int D_z I_z \mathrm{d}z \qquad \textit{Eq. S3}$$

For example, if static gas is ionized by the laser instead of a molecular beam pulse, the shape functions D_x, D_y and D_z are replaced by 1. The number of ionized molecules is then given by the laser ionization profile and the constant background pressure. However, if a molecular beam pulse is characterized, the number of ions in a volume element dxdydz is proportional to both the laser ionization probability and the molecule density in the volume element. We assume that the laser ionization probability is independent of molecular density.

The molecular density in the beam was determined through calibration of the ion detector with a static background gas of known density. Eq. S4 gives the ratio of ionized molecules in the molecular beam and in the calibration with the static gas

$$\frac{N^{\mathrm{MB}}}{N^{\mathrm{BG}}} = \frac{D_{\mathrm{max}}^{\mathrm{MB}} I_{\mathrm{max}} \int D_x I_x \mathrm{d}x \int D_y I_y \mathrm{d}y \int D_z I_z \mathrm{d}z}{D^{\mathrm{BG}} I_{\mathrm{max}} \int I_x \mathrm{d}x \int I_y \mathrm{d}y \int I_z \mathrm{d}z}. \qquad \textit{Eq. S4}$$

The number of ionized molecules using static gas does not include D_x, D_y and D_z as the density D^{BG} is constant in space. The absolute ionization probability of the laser I_{max} cancels, as the same laser intensity was used for molecular beam and static gas ionization. The laser focus is small compared to the molecular beam shape in directions orthogonal to the propagation axis (x and z), therefore $\int D_x I_x \mathrm{d}x \approx \tilde{D}_x \int I_x \mathrm{d}x$ with $\tilde{D}_x$ being the constant value which applies for the whole relevant integration

range. Experimentally, the laser focus is placed in the center of the beam such that $\tilde{D}_x = \tilde{D}_z = 1$. For D_y this cannot be done as the laser focus and molecular beam are of comparable size along the y-axis. From Eq. S4 we obtain

$$D_{\max}^{\mathrm{MB}} = D^{\mathrm{BG}} \frac{N^{\mathrm{MB}} \int I_y \mathrm{d}y}{N^{\mathrm{BG}} \int D_y I_y \mathrm{d}y}. \qquad \textit{Eq. S5}$$

To obtain the particle density of the molecular beam at its center $D_{\max}^{\mathrm{MB}}$, the density in the static gas experiment D^{BG} needs to be known. It was obtained from the N_2 equivalent ion gauge reading p_{IG}, which was converted to $H_2(D_2)$ pressure using the factor $g_{\mathrm{H_2(D_2)}} = 2.17(2.86)$.[3] Hydrogen was supplied to the chamber by a leak valve. The established constant background pressure was used to determine D^{BG} from the ideal gas law

$$D^{\mathrm{BG}} = \frac{p_{\mathrm{IG}} g_{\mathrm{H_2}}}{k_{\mathrm{B}} T} \qquad \textit{Eq. S6}$$

where k_{B} is Boltzmann's constant and T is the absolute temperature in the chamber (296 K). The ratio of produced ions when the laser hits the center of the molecular beam and when static gas is used is obtained as the experimental signal intensity ratio $N^{\mathrm{MB}}/N^{\mathrm{BG}} = C^{\mathrm{MB}}/C^{\mathrm{BG}}$ where C is the number of counts measured in arbitrary units.

The laser ionization function I_y is obtained by comparison of the beam shape function D_y and the ionized fraction of the molecular beam profile $D_y I_y$ as

$$I_y = \frac{D_y I_y}{D_y} \qquad \textit{Eq. S7}$$

The beam shape function D_y is obtained from an image, where ion signal was integrated while the laser focus is translated along the y-axis. This leads to a spatially homogeneous ionization efficiency, which reflects the molecular beam density shape on the ion image only. The quantity $D_y I_y$ is the shape resulting from the laser focus positioned in the center of the molecular beam. We emphasize that only the shape of the quantities $D_y I_y$, D_y and I_y is of importance.

With all these quantities in hand, Eq. S5 can be evaluated to yield $D_{\max}^{\mathrm{MB}}$ (molecules/m^3). In order to obtain the number of molecules within one beam pulse Eq. S1 needs to be integrated over the entire space. Two more transformations are employed to evaluate the integral. i) The integration along x (molecular beam propagation axis) is obtained by scanning the beam-laser delay time at fixed x-

position of the laser focus. This leads to the temporal shape function D_t^{MB}. The mean velocity of the molecular beam v_{MB} is used to convert time to space. ii) The beam shape along z cannot be measured in our detection geometry. However, radial symmetry can be assumed around the x-axis, as the beam is produced from a round orifice and passes a round skimmer. Integration in the xy-plane is therefore performed after transformation into polar coordinates, taking y as the radius. Finally, Eq. S8 results for the total number of molecules in a molecular beam pulse.

$$N = D_{\max}^{\mathrm{MB}} \int D_x \mathrm{d}x \int D_y \mathrm{d}y \int D_z \mathrm{d}z = D_{\max}^{\mathrm{MB}} \int v_{\mathrm{MB}} D_t^{\mathrm{MB}} \mathrm{d}t \int_0^{\infty} 2\pi y D_y \mathrm{d}y \qquad \textit{Eq. S8}$$

The number of molecules within a molecular beam pulse N is distributed over the surface according to the molecular beam shape function $D_y D_z$. When the dimension of propagation x or t is integrated, the exposed amount of H_2 molecules is given as:

$$[\mathrm{H_2}](y,z) = \frac{N}{2\pi\sigma^2\beta} D_y D_z \qquad \textit{Eq. S9}$$

where σ is the width of the molecular beam in y or z and $\beta(= 1.53 \times 10^{15}\mathrm{cm}^{-2})$ is the density of surface atoms. This leads to a dosage of H_2 molecules per surface atom. In principle, each point in the yz-plane leads to recombination with different H starting concentrations. This causes deviations from the rate expected for recombination from a single concentration pool. We investigated the effect of multiple initial concentrations by explicit simulation of kinetic traces in radial slices according to the spatial distribution. We found that the initial concentration distribution can be accounted for by using an effective exposure of $[\mathrm{H_2}]_{\mathrm{eff.}} = 0.5([\mathrm{H_2}](0{,}0) + [\mathrm{H_2}](2\sigma{,}0))$. This approximation allowed efficient evaluation of kinetic traces during the fitting procedure.

Finally, $[\mathrm{H_2}]_{\mathrm{eff.}}$ is distributed over the temporal profile of the incident beam to yield the time dependent H_2 flux:

$$F_t^{\mathrm{HH}} = \frac{D_t^{\mathrm{MB}} [\mathrm{H_2}]_{\mathrm{eff.}}}{\int D_t^{\mathrm{MB}} \mathrm{d}t} \qquad \textit{Eq. S10}$$

The time-dependent adsorbing flux of H atoms resulting from the exposure of H_2 is then given by $2S_0^{\mathrm{HH}} F_t^{\mathrm{HH}}$ with S_0 as the dissociative sticking coefficient. This expression can be found in Eq. S1 of the main text.

Table S1: Results for the effective exposure derived from the flux calibration for different H_2:D_2 mixing ratios of the molecular beam.

Mixing ratio (H_2:D_2)	$[H_2]_{eff.}$ [10^{-3} ML]	$[D_2]_{eff.}$ [10^{-3} ML]
50:50	0.69	2.06
75:25	1.35	1.95
90:10	1.57	0.71

2. Coarse Grained Grid Diffusion

It is well known that hydrogen atoms easily penetrate into the bulk of Pd metal. Using the well-known diffusion constant for hydrogen in bulk Pd,[4-7] a diffusion length on the order of 1 to 10 µm after 1 ms is expected, which is the typical timescale of the transient response in the present experiments. A diffusion length of 5 µm corresponds to about 20000 single (111)-layers of Pd, making it unpractical to simulate hopping between individual atomic layers. Therefore, we employ a coarse-grained grid to describe the spatial evolution of the concentration profile, see details below.

Close to the surface, individual (111) layers are simulated because in this region the biggest concentration gradients are expected. With increasing depth, gradients of H and D atom concentrations decrease, such that it is no more necessary to simulate the concentration of every single layer. Instead, the concentration evolution is simulated in a grid with exponentially growing grid point separation with increasing depth in the crystal, see Figure S1. The correct description of the concentration exchange between a grid point and its neighbours is derived from the diffusion equation. In general, the 1D diffusion equation along the spatial axis z is given as

$$\frac{\mathrm{d}c}{\mathrm{d}t} = D\frac{\mathrm{d}^2c}{\mathrm{d}z^2} \qquad \textit{Eq. S11}$$

where c is the concentration and D is the diffusion coefficient. Evaluation of the 2nd derivative of the concentration at spatial point z_i with concentration c_i is approximated by finite differences using the left and right neighbouring points.[8]

$$\left.\frac{\mathrm{d}^2c}{\mathrm{d}z^2}\right|_i = \frac{h_-c_{i+1} - (h_+ + h_-)c_i + h_+c_{i-1}}{\frac{1}{2}h_+h_-(h_+ + h_-)} \qquad h_+ = z_{i+1} - z_i \qquad h_- = z_i - z_{i-1} \qquad \textit{Eq. S12}$$

Further, the diffusion coefficient is replaced by its microscopic representation in one dimension: $D = \Gamma\lambda^2/2$. Here, Γ is the total jump frequency for a particle located in a discrete site. The site is connected to a left and a right neighbour by the jump length λ. Γ is composed of two equivalent contributions for hopping to the left k_l and to the right k_r. As k_bulk describes the transition from one site to the next in one direction, it follows: $\Gamma = 2k_\mathrm{bulk}$ and $D = k_\mathrm{bulk}\lambda^2$. Finally, the position variable z is expressed in units of the jump length by substituting $z_i = n_i \times \lambda$, where n_i is the number of single layers needed to reach the depth at point z_i. With $\left.\frac{\mathrm{d}^2c}{\mathrm{d}n^2}\right|_i = \left.\frac{\mathrm{d}^2c}{\mathrm{d}z^2}\right|_i \left(\left.\frac{\mathrm{d}z}{\mathrm{d}n}\right|_i\right)^2$, the diffusion equation is written as

$$\left.\frac{\mathrm{d}c}{\mathrm{d}t}\right|_i = k_\mathrm{bulk}\left.\frac{\mathrm{d}^2c}{\mathrm{d}n^2}\right|_i \qquad \textit{Eq. S13}$$

In summary, Eq. S13 evaluates the rate of concentration change at grid point i, where the spatial axis is given as depth below the surface in units of the single jump length. If grid points are separated by the (111) layer-width, the diffusion equation turns into the kinetic equation for explicit hopping in a uniform grid. Eq. S14 - Eq. S16 represent the full set of differential equations for H used to propagate the model in time. Analogously, equations for D are implemented. Typically, the grid consists of about 100 points (compare Figure S1).

$$\frac{\mathrm{d}[\mathrm{H}_0]}{\mathrm{d}t} = 2S_0^\mathrm{HH}F_t^\mathrm{HH} - 2k_\mathrm{rec}^\mathrm{HH}[\mathrm{H}_0]^2 - k_\mathrm{rec}^\mathrm{HD}[\mathrm{H}_0][\mathrm{D}_0] - k_\mathrm{bulk}^\mathrm{H}K^\mathrm{H}[\mathrm{H}_0] + k_\mathrm{bulk}^\mathrm{H}[\mathrm{H}_1] \qquad \textit{Eq. S14}$$

$$\frac{\mathrm{d}[\mathrm{H}_1]}{\mathrm{d}t} = k_\mathrm{bulk}^\mathrm{H}K^\mathrm{H}[\mathrm{H}_0] - 2k_\mathrm{bulk}[\mathrm{H}_1] + k_\mathrm{bulk}[\mathrm{H}_2] \qquad \textit{Eq. S15}$$

$$\frac{\mathrm{d}[\mathrm{H}_i]}{\mathrm{d}t} = k_\mathrm{bulk}^\mathrm{H} \times \left.\frac{\mathrm{d}^2[\mathrm{H}_i]}{\mathrm{d}n^2}\right|_i \text{ for } i > 1 \qquad \textit{Eq. S16}$$

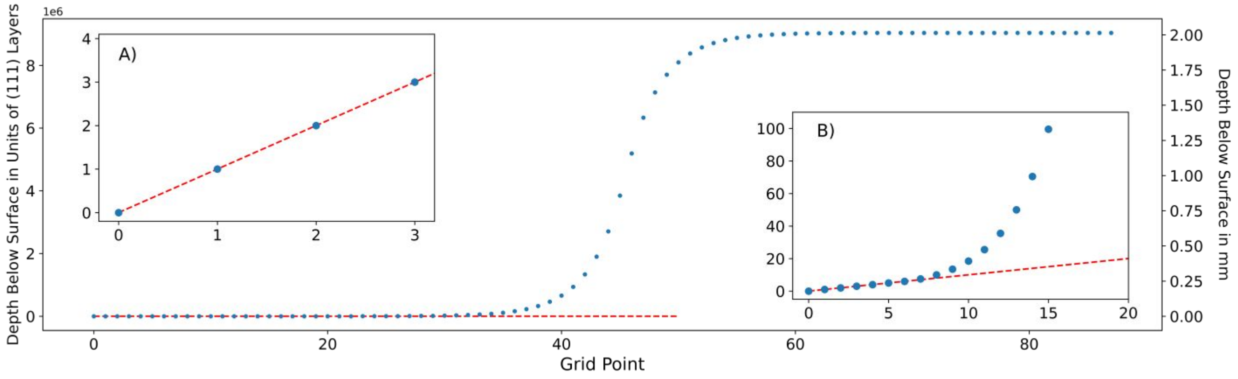

Figure S1: Exponential grid used to simulate the whole Pd crystal. The surface is located at gridpoint 0, the 1st subsurface site at 1 and so on. The y-axis shows the depth below the surface. Close to the surface, grid points are separated the (111) layer spacing, see inset A). Deeper inside the crystal, the distance between grid points increases exponentially until the center of the crystal is reached. Inset B) shows the transition from linear to exponential spacing. Then, the grid point distance is decreased again symmetrically until the other side of the crystal is reached at 2 mm.

3. Bulk Diffusion Constants for H and D

The bulk diffusion constant was obtained from an Arrhenius fit to experimental data from literature[5-7] (see Figure S2). The fit yields the diffusion barrier E_{diff} and the pre-factor D_0.

$$D = D_0 \exp\left(-\frac{E_{\text{diff}}}{k_{\text{B}}T}\right) \qquad \textit{Eq. S17}$$

The microscopic hopping pre-factor A is obtained from the macroscopic D_0 as

$$A = \frac{D_0}{\lambda^2} \qquad \textit{Eq. S18}$$

where λ is the distance between two (111) layers, which relates to the lattice constant of palladium $a = 389$ pm[9] as $\lambda = a/\sqrt{3}$.

The resulting parameters are listed in Table S2:

Table S2: Arrhenius parameters for H and D hopping between two (111)-layers in bulk Pd.

	$A\ [10^{12}\text{s}^{-1}]$	E_{diff} [eV]
H	6.094	0.232
D	4.272	0.215

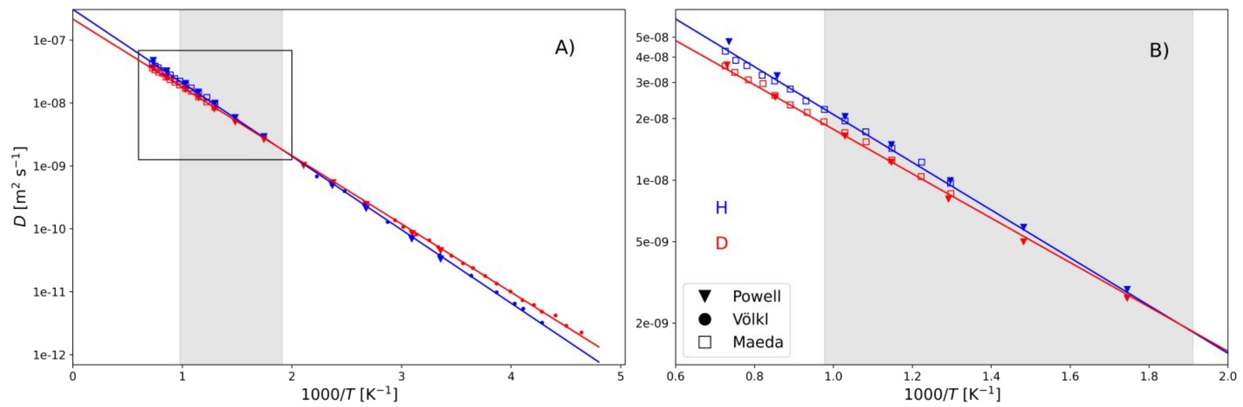

Figure S2: Diffusion constants for H and D in palladium. A) shows the full temperature range and B) shows the magnification indicated by the rectangle in A). Symbols represent literature data from Powell[6], Völkl[5] and Maeda[7]. Solid lines are Arrhenius fits to the combined literature data. The grey shaded region marks the temperature range of this work. Blue and red lines and symbols correspond to H and D, respectively.

4. Bulk Potential and Partition Function

The effective potential used in the desorption/diffusion model for an H atom absorbed in the bulk of Pd $V_{\mathrm{bulk}}(x)$ is shown in Figure S3 and is described by Eq. S19.

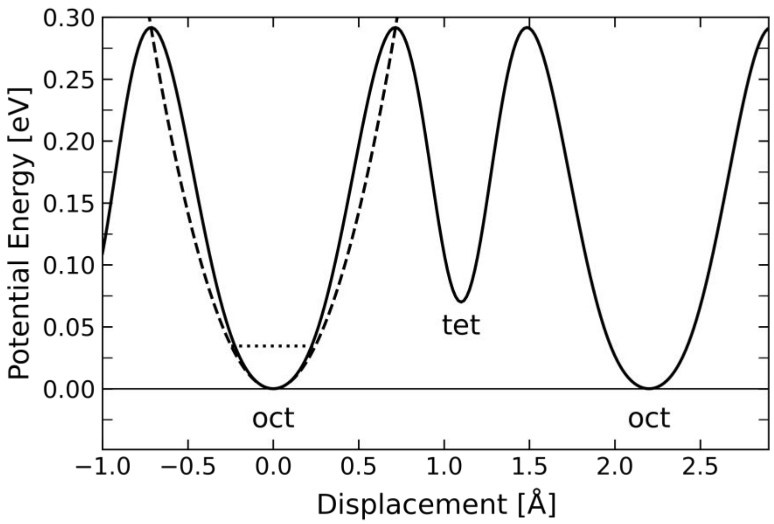

Figure S3: One dimensional potential energy curve for a bulk absorbed H atom. Octahedral (oct) and tetrahedral (tet) binding sites are labelled. The dashed curve represents a harmonic oscillator with a frequency equivalent to the harmonic frequency of the potential at its minimum and the dotted line indicates the harmonic oscillator's ground state.

$$V_{\mathrm{bulk}}(x) = w \times \left((1-g) \times \left(1 - \cos^4 \frac{\pi x}{2.2\text{Å}}\right) + g \times \left(1 - \cos^4 \frac{\pi x}{2.2\text{Å}}\right)^2 \right) + f \cos^{14} \frac{\pi(x - 1.1\text{Å})}{2.2\text{Å}}$$

Eq. S19

where the optimized values of the parameters were

$$w = 0.3664 \text{ eV};\ g = 0.6190; f = -0.2964 \text{ eV}$$

Note that the potential function was obtained by fitting the absorption enthalpy data from previous work[10] together with the present kinetic traces as described in the main text. In the fitting procedure, we forced the harmonic frequency, calculated from the minimum energy position of the octahedral site (dashed line Figure S3), to the experimental value of 0.069 eV, that has been measured by neutron scattering.[11] This treatment guarantees an accurate ZPE and anchors the potential to measured quantities. The best fit curve obtained here is highly anharmonic and its properties are consistent with conclusions drawn from neutron scattering studies.[11] Further, we also incorporated a site that mimics the tetrahedral site. At high energy, the free particle limit is approached. This description features physical anchor points and is capable to effectively fit the experimental observations.

The partition function of V_{bulk} is the most critical quantity to describe the temperature dependence of the absorption enthalpy as well as being important to describe the surface to bulk transition rate. It is obtained from the classical partition function corrected for low temperature quantum effects using the Pitzer-Gwinn correction.[12, 13] The three-dimensional partition function is thus given as

$$Q_{\mathrm{H}}^{\mathrm{bulk}} = \left(q_{\mathrm{H}}^{\mathrm{bulk\ 1D}}\right)^3 = \left(\frac{2\pi m k_{\mathrm{B}} T}{h^2}\right)^{\frac{3}{2}} \times \left(\int \exp\left(-\frac{V_{\mathrm{bulk}}(x)}{k_{\mathrm{B}} T}\right) \mathrm{d}x \times \frac{q_{\mathrm{vib}}^{\mathrm{qho}}}{q_{\mathrm{vib}}^{\mathrm{cho}}} \right)^3 \qquad \textit{Eq. S20}$$

5. Modelling the Absorption Enthalpy

The absorption enthalpy $\Delta H_{\mathrm{abs}}^{\mathrm{H_2}}$ at given temperature can be determined from the absorption energy $\epsilon_{\mathrm{abs}} + \Delta\mathrm{ZPE}$ and the temperature dependent internal energy difference $\Delta U(T)$, following:

$$\Delta H_{\mathrm{abs}}^{\mathrm{H_2}} = \epsilon_{\mathrm{abs}} + \Delta\mathrm{ZPE} + \Delta U(T) - k_{\mathrm{B}} T \qquad \textit{Eq. S21}$$

with $\Delta\mathrm{ZPE} = 2 \cdot \mathrm{ZPE}_{\mathrm{abs}}^{\mathrm{H}} - \mathrm{ZPE}_{\mathrm{gas}}^{\mathrm{H_2}}$ and $\Delta U(T) = 2 \cdot U_{\mathrm{bulk}}^{\mathrm{H}}(T) - U_{\mathrm{gas}}^{\mathrm{H_2}}(T)$ where

$$U_{\mathrm{bulk}}^{\mathrm{H}}(T) = k_{\mathrm{B}}T^2\frac{\partial \ln Q_{\mathrm{bulk}}^{\mathrm{H}}}{\partial T}$$

and *Eq. S22*

$$U_{\mathrm{gas}}^{\mathrm{H_2}}(T) = k_{\mathrm{B}}T^2\frac{\partial \ln Q_{\mathrm{gas}}^{\mathrm{H_2}}}{\partial T}$$

The construction in Eq. S21 considers first the classical energy difference between H_2 and 2H(bulk), then corrects for zero-point vibrational energy, then adds thermal internal energy and finally subtracts $k_{\mathrm{B}}T$ to convert to enthalpy as a gas phase molecule is consumed during absorption. The partition functions $Q_{\mathrm{bulk}}^{\mathrm{H}}$ and $Q_{\mathrm{gas}}^{\mathrm{H_2}}$ are defined in SI sec. 4 and 7, respectively.

The absorption enthalpy is shown as a function of temperature in Figure 3 of the main paper.

6. Thermal Sticking Coefficient

Thermal sticking coefficients of H_2 and D_2 were obtained by averaging energy dependent sticking coefficients measured at the surface normal.[14, 15] The applied procedure is described in Ref.[1]. Figure S4 shows the result. The thermal sticking coefficient of HD was assumed to be the average of the values for H_2 and D_2. We obtained a temperature independent value of $\mathrm{S}_0^{\mathrm{HD}} = 0.535$.

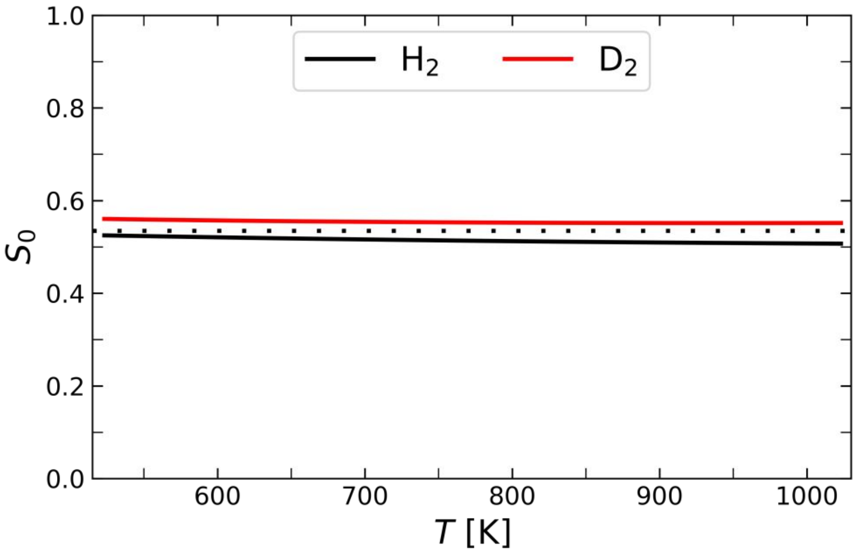

Figure S4: Thermal sticking coefficient S_0 of H_2 and D_2 extracted according to description in the text. The dotted line is the average of the values of H_2 and D_2 and is used as $S_0^{HD} = 0.535$ in the present kinetic model.

7. H(D)-Recombination Rate Constant

The hydrogen atom recombination rate constant is given by Eq. S8 of the main text. The specific contributions to this formula are described in the following. For the hydrogen atom partition function $Q_\mathrm{A}^\mathrm{ads}$ (Eq. S23), separability of motion in the xy-plane from motion along the z-coordinate is assumed. Besides the atom motion, also an electronic part of the partition function has to be included as each adsorbed atom carries two degenerate spin-states ($q_\mathrm{A}^\mathrm{el} = 2$). The four resulting spin-states are not degenerate in the product molecule anymore. It will only be formed in its singlet electronic ground-state.

$$Q_\mathrm{A}^\mathrm{ads} = \frac{q_\mathrm{A}^{xy}}{A_\mathrm{ref}} \times \bar{q}_\mathrm{A}^{z} \times q_\mathrm{A}^\mathrm{el} \qquad \textit{Eq. S23}$$

The in-plane partition function q_A^{xy} was obtained by solving the single particle Schödinger equation for H or D on the 2D in-plane interaction potential and was divided by the area of the periodic cell A_ref. The in-plane partition function results from the energy eigenvalues E_i as

$$q_\mathrm{A}^{xy} = \sum_{i=0}^{n} \exp\left(-\frac{E_i - E_0}{k_\mathrm{B}T}\right) \qquad \textit{Eq. S24}$$

where E_0 is the ground-state energy and n is chosen such that the partition function is converged for a given temperature. The procedure is described in great detail in Ref.[1]. The in-plane H-Pd interaction PES was obtained from DFT by calculating a representative xy-energy grid for each surface (see SI sec. 14). The z-coordinate (out of plane) of the atom was held at the minimum energy at each xy point. This assumes that in-plane and out of plane movement are decoupled. Figure S5 shows the interpolated interaction potentials for H on Pd(111) and Pd(332) with the zero-point energy of the z-coordinate added.

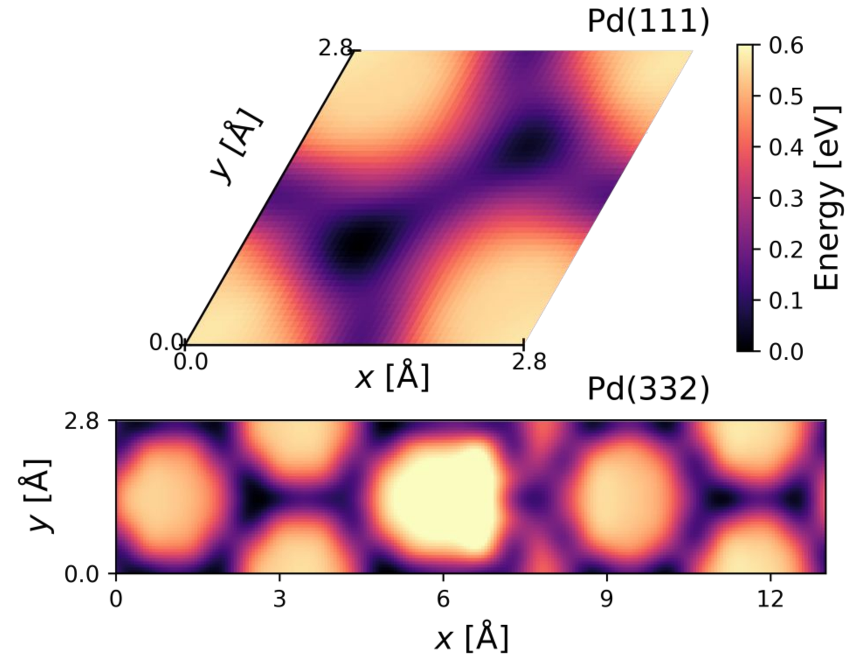

Figure S5: In-plane potential energy surface for H/Pd(111) and H/Pd(332) with ZPE of the H-Pd stretch vibration added.

The H(D)-Pd stretch vibration is assumed harmonic, whereby the frequency is determined from our DFT calculations. The H-Pd stretch frequencies depend on the in-plane position of the H atom. For example the z-stretch frequency is 0.1256 eV on the fcc site and 0.1539 eV on the bridge site for H/Pd(111). Therefore, we need to account for the coupling of z-stretch frequency to the in-plane coordinates.[16] The xy-dependent frequencies $\nu_{\mathrm{PdA}}(x,y)$ are used to obtain the mean partition function $\bar{q}_{\mathrm{A}}^{z}$. This quantity is the spatial average of the Pd — A vibrational partition function, weighted by the thermal probability density of the H atom in the xy-plane:

$$\bar{q}_{\mathrm{A}}^{z} = \frac{\sum_i \int p_i \times \left|\Psi_i^2(x,y)\right| \times q_{\mathrm{A}}^{z}(x,y)\mathrm{d}x\mathrm{d}y}{\sum_i \int p_i \times \left|\Psi_i^2(x,y)\right| \mathrm{d}x\mathrm{d}y}$$

with

Eq. S25

$$q_{\mathrm{A}}^{z}(x,y) = \left(1 - \exp\left(-\frac{h\nu_{\mathrm{PdA}}(x,y)}{k_{\mathrm{B}}T}\right)\right)^{-1}$$

and

$$p_i = \exp\left(-\frac{E_i - E_0}{k_\mathrm{B}T}\right)$$

Here, p_i is the thermal population of quantum state i for H in the xy-plane and $\left|\Psi_i^2(x,y)\right|$ is the probability density function of state i.

The partition function of the gas phase product molecule is given as

$$Q_\mathrm{AB}^\mathrm{gas} = \frac{q_\mathrm{AB}^\mathrm{trans\ 3D}}{V_\mathrm{ref}} \times q_\mathrm{AB}^\mathrm{rot} \times q_\mathrm{AB}^\mathrm{vib} \times q_\mathrm{AB}^\mathrm{el} \qquad \textit{Eq. S26}$$

$$= \left(\frac{\sqrt{2\pi m_\mathrm{AB} k_\mathrm{B} T}}{h}\right)^3 \times \frac{1}{\sigma_\mathrm{AB}} \frac{k_\mathrm{B}T}{B_\mathrm{AB}} \times \frac{1}{1 - \exp\left(-\frac{h\nu_\mathrm{AB}}{k_\mathrm{B}T}\right)} \times 1.$$

In Eq. S26 m_{AB} denotes the molecular mass, σ_{AB} is the symmetry number, B_{AB} is the rotational constant in the vibrational ground-state and ν_{AB} is the vibrational frequency of the molecule. Required molecular constants were taken from experiments and are reported in Table S4.

The partition functions were defined to start from the lowest quantum state and not from the classical minimum of the PES. Therefore, the binding energy E_0^AB includes the zero-point energies. It thus depends on the recombining isotopologues:

$$E_0^\mathrm{AB} = \epsilon_\mathrm{rec} + \mathrm{ZPE}_\mathrm{AB}^\mathrm{gas} - \left(\mathrm{ZPE}_\mathrm{A}^\mathrm{surf} + \mathrm{ZPE}_\mathrm{B}^\mathrm{surf}\right). \qquad \textit{Eq. S27}$$

In Eq. S27 ϵ_rec denotes the classical dissociative binding energy of hydrogen at the surface. Table S3 summarizes the zero-point energies of the adsorbed H-atom.

Table S3: Zero-point-energies for interaction of A with Pd(111) or Pd(332). ZPE_A^{xy} is the ground-state energy of the 2D in-plane PES (see text) and ZPE_A^{z} is half the harmonic frequency of the vibration along the z-coordinate at the minimum of the z-ZPE-added PES. The total ZPE of the hydrogen atom is $ZPE_A^{surf} = ZPE_A^{xy} + ZPE_A^{z}$.

	Pd(111)	Pd(332)

A	$\mathrm{ZPE}_\mathrm{A}^{xy}$ [eV]	$\mathrm{ZPE}_\mathrm{A}^{z}$ [eV]	$\mathrm{ZPE}_\mathrm{A}^{xy}$ [eV]	$\mathrm{ZPE}_\mathrm{A}^{z}$ [eV]
H	0.102 (0.0888)[a)]	0.1256	0.0869	0.152
D	0.074 (0.0633) [a)]	0.0888	0.0611	0.107

[a)] Values in parentheses are obtained from the RPBE PES of Ref. [17]. These are used for the uncertainty evaluation.

Table S4: Used experimental molecular constants with ν_{AB} being the stretching frequency, B_{AB} being the rotational constant in the vibrational ground state and σ_{AB} being the symmetry number of rotation. Values were taken from NIST Chemistry WebBook.[18]

AB	ν_AB [eV]	B_AB [10^{-3} eV]	σ_AB
HD	0.47276	5.5374	1
HH	0.54568	7.3550	2
DD	0.38627	3.7076	2

8. Uncertainty Range

The uncertainty was evaluated by fitting the model parameters using i) a H_2/D_2 dose increased or decreased by 30% and ii) the in-plane partition function and ZPE from either the present PBE PES or the RPBE PES from Ref. [17] for Pd(111). From Table S5, we estimate $\epsilon_\mathrm{ads} = 1.02 \pm 0.03$ eV and $\epsilon_\mathrm{abs} = -0.093 \pm 0.005$ eV.

Table S5: Best fit parameters of the model to the present kinetic data and absorption enthalpy data as presented in the main text. The uncertainty range is estimated by either using PBE or RPBE to construct the in-plane PES for H/Pd(111). Further, the incident dose to initiate the reaction was increased or decreased by 30%.

Functional	Dose	ϵ_ads [eV]	ϵ_abs [eV]	w [eV]	g	f [eV]
PBE	130%	1.053	−0.097	0.3451	0.5954	−0.2821
PBE	100%	1.039	−0.093	0.3664	0.6190	−0.2964

PBE	70%	1.019	−0.087	0.3939	0.6455	−0.3120
RPBE	130%	1.016	−0.097	0.3456	0.5960	−0.2824
RPBE	100%	1.001	−0.093	0.3673	0.6199	−0.2974
RPBE	70%	0.982	−0.087	0.3940	0.6457	−0.3119

9. Velocity Resolved Kinetics Data of Pd(332)

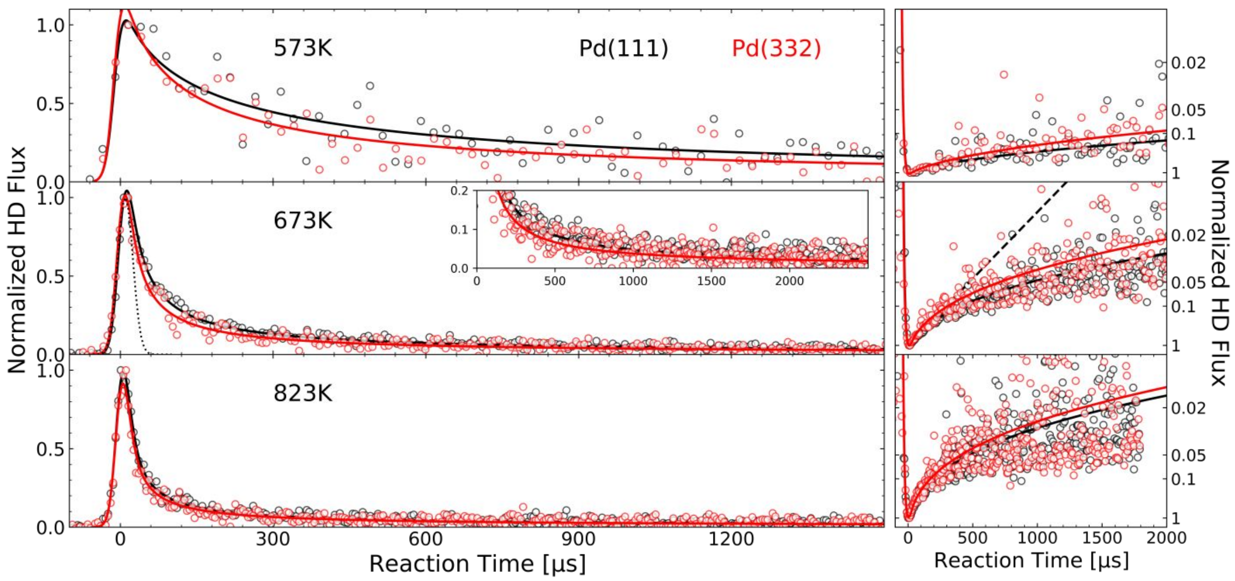

Figure S6: HD formation rate as a function of reaction time on Pd(111) (empty black circles) and Pd(332) (empty red circles). The time dependence of the dosing pulse is shown as a dotted line. H_2 and D_2 were dosed with 1.35 and $1.95 \times 10^{-3} ML$ per pulse, respectively. The solid lines show the best fit of the kinetic model involving recombinative desorption and diffusion into the bulk. The right column panels show the data after attempted linearization. The dashed line shows the behavior expected from a 2nd order reaction.

10. Temperature Dependent Rate Constants as Extended Arrhenius

To provide easy access to the isotope-specific rate constants, we parametrized the present model for recombination (Eq. S8 of the main paper) by fitting an extended Arrhenius equation (Eq. S28) to it in a temperature range from 300 to 1100 K. The extended Arrhenius fit shows typical deviations <1% with respect to the full model. The maximum deviation is 3%. The results are provided in Table S6.

$$k(T) = \alpha \times \left(\frac{T}{\mathrm{K}}\right)^{\beta} \times \exp\left(-\frac{\gamma}{k_\mathrm{B}T}\right) \qquad \textit{Eq. S28}$$

The equilibrium constant $K^\mathrm{A} = k_{01}^\mathrm{A}/k_\mathrm{bulk}^\mathrm{A}$ was parametrized with the same formula. Deviations do not exceed 3%. Table S7 shows the result. Together with the diffusion constant $k_\mathrm{bulk}^\mathrm{A}$ (see SI sec. 3), the artificial rate constant k_{01}^A can be obtained as well. Notice that only K^A and $k_\mathrm{bulk}^\mathrm{A}$ are real physical quantities.

Table S6: Parameters for extended Arrhenius rate expression of the recombinative desorption rate constants.

AB	α [(MLs) $^{-1}$]	β	γ [eV]
HH	2.886×10^{19}	-1.909	1.085
HD	1.389×10^{21}	-2.353	1.097
DD	1.455×10^{22}	-2.778	1.101

Table S7: Parameters for $K^A(T)$.

A	α	β	γ [eV]
H	5.747×10^{-3}	0.729	0.393
D	3.828×10^{-2}	0.490	0.419

11. Reanalysis of Conrad *et al.* Isotherms

In order to quantify the recombination rate constants which underlie the work function isotherm data of Conrad *et al.*[19] an Arrhenius expression was used to simulate their data. In these experiments all coverages between 1 ML and 0 will be present depending on temperature and H_2 pressure. This complicates the extraction of zero coverage rate constants as the apparent coverage dependence of the rate constant due to adsorbate-adsorbate interactions must be accounted for as well. Here, the coverage dependent rate constant for hydrogen recombination was modeled using the Elovich expression[20]:

$$k(T,[\mathrm{H}]) = A \exp\left(-\frac{E_\mathrm{a} - \alpha[\mathrm{H}]}{k_\mathrm{B}T}\right) \qquad \textit{Eq. S29}$$

Isotherms were simulated by propagating the following differential equation in time, until a steady state is reached.

$$\frac{\mathrm{d[H]}}{\mathrm{d}t} = 2S_0^{\mathrm{HH}}\left(1-\frac{\mathrm{[H]}}{1\ \mathrm{ML}}\right)^2 \frac{p}{\sqrt{2\pi m k_\mathrm{B} T}} - 2k(T,\mathrm{[H]})\ \mathrm{[H]}^2 \qquad \textit{Eq. S30}$$

Inclusion of bulk population does not affect the result and is not considered in this simulation. The resulting data of steady state hydrogen coverage at given temperature and pressure are compared to the experimental isotherms in Figure S7. A global amplitude parameter is used to transfer work function change to coverage. This assumes a linear relationship between work function and coverage.

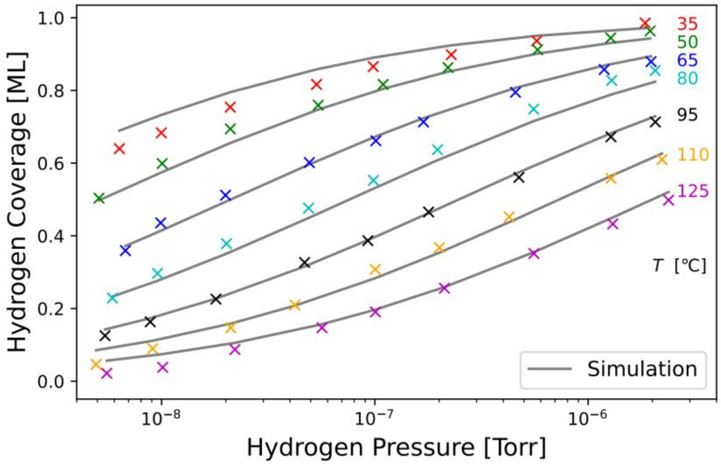

Figure S7: Fitting of adsorption Isotherms of Conrad et al.[19]

The best fit parameters are $E_\mathrm{a} = 0.89$ eV, $A = 1.3 \times 10^{11}$ (MLs)$^{-1}$ and $\alpha = 0.02\frac{\mathrm{eV}}{\mathrm{ML}}$. The small coverage dependence is consistent with findings of Conrad *et al.*[19]

12. Reanalysis of Gdowski *et al*. TPD Data

In order to quantify the recombination rate constants which underlie the TPD data of Gdowski *et al.*[21] an Arrhenius expression was used to simulate their data. In TPD experiments all coverages between ~1 ML and 0 occur during the temperature ramp. Therefore, Eq. S29 was used here as well. The TPD data were simulated by propagating the following differential equation in time

$$\frac{\mathrm{d[H]}}{\mathrm{d}t} = -2k(T,\mathrm{[H]})\ \mathrm{[H]}^2 \qquad \textit{Eq. S31}$$

The simulation was started at an initial coverage $\mathrm{[H]}_0$, which was obtained by the integral of the experimental traces, whereby the coverage of the 3.45 L dosage (see Figure 1 of Ref. [21]) was set to 0.76 ML as the area of the TPD traces was not converged yet. The temperature at the beginning was set to 80 K and it increased with time according to the experimental heating rate $\beta = 5.8\frac{\mathrm{K}}{\mathrm{s}}$. The

simulation leads to the time evolution of the hydrogen coverage $[\mathrm{H}]_t$ and the TPD trace is obtained as the H_2 production rate $r = k[\mathrm{H}]_t{}^2$ and is plotted as a function of temperature using $T(t) = 80\mathrm{K} + \beta t$. A global amplitude parameter is used to transfer the arbitrary experimental rate to the absolute simulated rate. The resulting fits are shown in Figure S8.

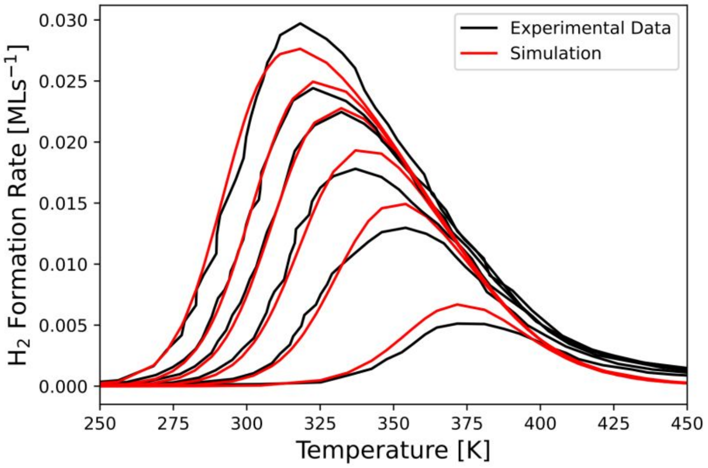

Figure S8: Fit to TPD data of Gdowski et al.[21]

The best fit parameters are $E_\mathrm{a} = 0.91$ eV, $A = 1.8 \times 10^{12}$ $(\mathrm{MLs})^{-1}$ and $\alpha = 0.165\frac{\mathrm{eV}}{\mathrm{ML}}$. The resulting coverage dependence of E_a agrees with the the study of Engel and Kuipers.[22]

13. Tracer Kinetic Monte Carlo (TkMC) Method

To visualize H atom bulk penetration in the recombinative desorption experiment, we implemented a single particle kMC method. This allows to track the maximum penetration depth (MPD) of the propagated atom and correlate it to its time of recombination. This leads to the results presented in Figure 5 B) of the main paper.

A single H atom was propagated in the recombination diffusion system according to the framework of kinetic Monte Carlo. The frequency of occurrence of any hopping process is given by the respective rate constant, as the process is 1st order. However, H atom recombination on the surface cannot be achieved by a single H atom. Therefore, an effective first order recombination rate is obtained by using $w_\mathrm{rec} = k_\mathrm{rec}^\mathrm{HH}[\mathrm{H}_0]_t$. This is the number of recombination events per second a single H atom would undergo at a given surface coverage $[\mathrm{H}_0]_t$. If an H atom is located on the surface at time t, the probability for it to recombine is $p_\mathrm{rec} = w_\mathrm{rec}/(w_\mathrm{rec} + w_{01})$, where

$$w_{\mathrm{rec}} = 2k_{\mathrm{rec}}^{\mathrm{HH}}[\mathrm{H}_0]_t \quad \text{and} \quad w_{01} = k_{01}^{\mathrm{H}} = k_{\mathrm{bulk}}^{\mathrm{H}} \times K^{\mathrm{H}} \qquad \textit{Eq. S32}$$

with w_{01} being the rate for an H atom to penetrate into the subsurface and w_{rec} being the rate of recombination with the mean-field H concentration. The sum of all possible rates is referred to as w_{tot}. The hydrogen concentration on the surface $[\mathrm{H}_0]_t$ is given by the mean-field simulation introduced in Sec. 2.B of the main paper.

The simulation proceeds as follows: For each trajectory, a t_0 is drawn from the experimental dosing flux distribution. Then, the trajectory is propagated as follows:

1) Make a list of possible processes for the H atom in the current position. For example, at t_0 these are recombination with the mean-field and hopping into the first subsurface.
2) Choose and execute a process according to principles of kMC[23] using random number r_1.
3) Update time according to principles of kMC[23] using random number r_2.
4) Store the grid point at which the H atom sits only if it corresponds to a new maximum depth.

Steps 1) to 4) are executed until the H atom undergoes recombination or the simulated time exceeds 2 ms. Figure S9 illustrates the propagation of a H atom. The simulation is carried out in a grid more coarsely grained than the one used for simulation of the mean-field kinetics to reduce simulation time.

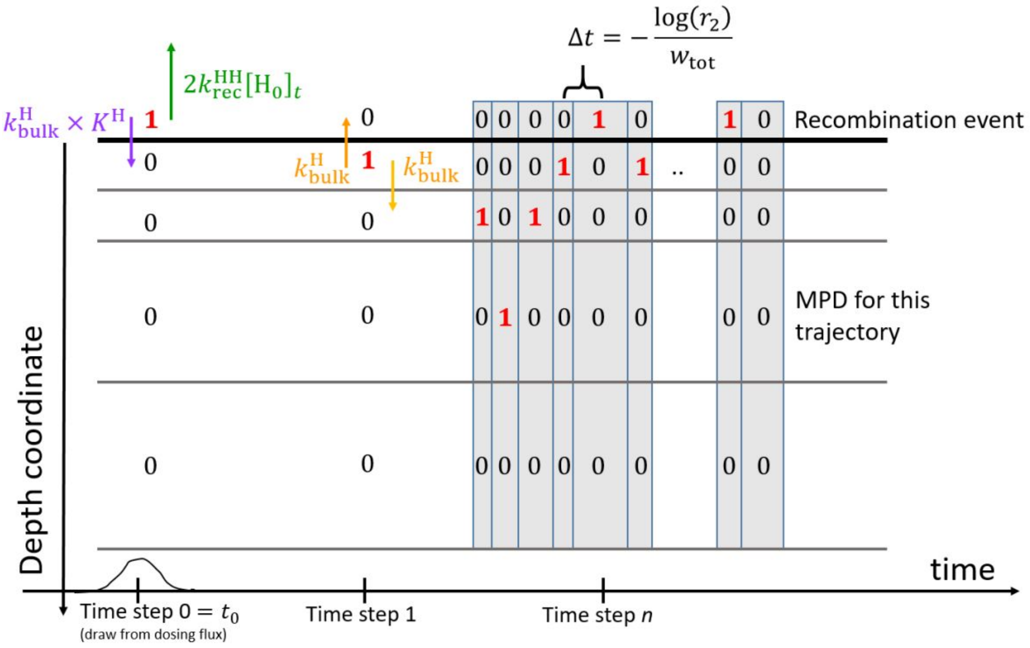

Figure S9: TkMC approach. The red 1 indicates that this site is occupied, while 0 indicates an empty site. For the first two time steps, the rates of the possible processes are marked in color. See text for further description.

14. Computational Details

The DFT calculations were performed with the Vienna ab-initio simulation package (VASP5.3.5).[24-27] For the description of electronic exchange and correlation effects, the PBE functional has been used for all calculations.[28, 29] The optimum lattice constant of palladium was found to be 3.934 Å. The Pd(111) surface is modelled by a 2×2 slab with 6 layers, whereas the two bottom layers were fixed. Relaxation of all other layers was performed until forces and energies per atom were smaller than 1 meV/Å and 0.01 meV, respectively. To prevent interactions of the slab with its periodic images, a vacuum layer with a thickness of 14 Å was applied. The Brillouin zone was sampled by a $10 \times 10 \times 1$ k-point grid making use of the sampling scheme proposed by Monkhorst and Pack.[30] For all calculations, the energy cut-off was set to 400 eV. The electronic energies of the H/Pd(111) PES were acquired by calculating 10 high symmetry points on the irreducible part of the surface. We permitted relaxation only of the H-atom's degree of freedom perpendicular to the surface plane (along the z-axis) and the structure optimization was stopped when forces < 0.01 eV/Å were reached. The optimized geometry served as a starting point for the calculation of the harmonic H(D)-Pd stretch frequency, which was determined from the dynamical matrix using a finite difference method with four displacements along the z-axis. The H(D)-Pd harmonic stretch frequencies were used for the z-axis ZPE correction, see SI sec. 7.

The Pd(332) surface was modelled as a $p(4 \times 1)$ slab with four layers. The same convergence criteria as for Pd(111) were applied for the relaxation of the slab. However, a $4 \times 4 \times 1$ k-point grid was used to represent the Brillouin zone. The Pd(332) PES was sampled by calculating interaction energies, arranged in a 5×28 equidistant grid along the irreducible part of the Pd(332) surface. The corresponding harmonic frequencies were calculated in the same manner as for Pd(111).

In addition, the absorption energy was calculated using the PBE and the RPBE[31] functional. We represented the Pd bulk as $2a_0 \times 2a_0 \times 2a_0$ super cell with a_0 being the optimal conventional lattice constant of the respective functional (PBE: 3.934 Å, RPBE: 3.975 Å). In order to acquire the absorption energy, we placed an H atom at the interstitials of interest and subsequently let the super structure relax, where the cell volume was subject to optimization too. For integration over the Brillouin-zone, we utilized a $10 \times 10 \times 10$ Monkhorst-Pack k-point mesh. The energy cutoff of the plane waves was set to 400 eV.

References

(1) Borodin, D.; Hertl, N.; Park, G. B.; Schwarzer, M.; Fingerhut, J.; Wang, Y.; Zuo, J.; Nitz, F.; Skoulatakis, G.; Kandratsenka, A.; et al. Quantum effects in thermal reaction rates at metal surfaces. *Science* **2022**, *377*, 394-398.

(2) Meng, C. S.; Janssen, M. H. M. Measurement of the density profile of pure and seeded molecular beams by femtosecond ion imaging. *Rev Sci Instrum* **2015**, *86*.

(3) *Stanford Research Systems, Gas Correction Factors for Bayard-Alpert Ionization Gauges, https://www.thinksrs.com/downloads/pdfs/applicationnotes/IG1BAgasapp.pdf, accessed on 21-06-2022*.

(4) Holleck, G. L. Diffusion and Solubility of Hydrogen in Palladium and Palladium-Silver Alloys. *J Phys Chem-Us* **1970**, *74*, 503-&.

(5) Volkl, J.; Wollenweber, G.; Klatt, K. H.; Alefeld, G. Reversed Isotope Dependence for Hydrogen Diffusion in Palladium. *Z Naturforsch Pt A* **1971**, *A 26*, 922-+.

(6) Powell, G. L.; Kirkpatrick, J. R. Surface Conductance and the Diffusion of H and D in Pd. *Phys Rev B* **1991**, *43*, 6968-6976.

(7) Maeda, T.; Naito, S.; Yamamoto, M.; Mabuchi, M.; Hashino, T. High-Temperature Diffusion of Hydrogen and Deuterium in Palladium. *J Chem Soc Faraday T* **1994**, *90*, 899-903.

(8) Veldman, A. E. P.; Rinzema, K. Playing with Nonuniform Grids. *J Eng Math* **1992**, *26*, 119-130.

(9) Arblaster, J. W. Crystallographic Properties of Palladium Assessment of properties from absolute zero to the melting point. *Platin Met Rev* **2012**, *56*, 181-189.

(10) Manchester, F. D.; San-Martin, A.; Pitre, J. M. The H-Pd (hydrogen-palladium) System. *Journal of Phase Equilibria* **1994**, *15*, 62-83.

(11) Rush, J. J.; Rowe, J. M.; Richter, D. Direct Determination of the Anharmonic Vibrational Potential for H in Pd. *Z Phys B Con Mat* **1984**, *55*, 283-286.

(12) Pitzer, K. S.; Gwinn, W. D. Energy Levels and Thermodynamic Functions for Molecules with Internal Rotation I. Rigid Frame with Attached Tops. *The Journal of Chemical Physics* **1942**, *10*, 428-440.

(13) Borodin, D.; Rahinov, I.; Fingerhut, J.; Schwarzer, M.; Horandl, S.; Skoulatakis, G.; Schwarzer, D.; Kitsopoulos, T. N.; Wodtke, A. M. NO Binding Energies to and Diffusion Barrier on Pd Obtained with Velocity-Resolved Kinetics. *J Phys Chem C* **2021**, *125*, 11773-11781.

(14) Resch, C.; Berger, H. F.; Rendulic, K. D.; Bertel, E. Adsorption Dynamics for the System Hydrogen/Palladium and Its Relation to the Surface Electronic-Structure. *Surf Sci* **1994**, *316*, L1105-L1109.

(15) Kratzer, M.; Stettner, J.; Winkler, A. Angular distribution of desorbing/permeating deuterium from modified Pd(111) surfaces. *Surf Sci* **2007**, *601*, 3456-3463.

(16) Borodin, D.; Rahinov, I.; Galparsoro, O.; Fingerhut, J.; Schwarzer, M.; Golibrzuch, K.; Skoulatakis, G.; Auerbach, D. J.; Kandratsenka, A.; Schwarzer, D.; et al. Kinetics of NH3 Desorption and Diffusion on Pt: Implications for the Ostwald Process. *J Am Chem Soc* **2021**, *143*, 18305-18316.

(17) Kristinsdottir, L.; Skulason, E. A systematic DFT study of hydrogen diffusion on transition metal surfaces. *Surf Sci* **2012**, *606*, 1400-1404.

(18) *NIST Chemistry WebBook, https://webbook.nist.gov/cgi/cbook.cgi?Name=hydrogen&Units=SI, accessed on 28-06-2022*.

(19) Conrad, H.; Ertl, G.; Latta, E. E. Adsorption of Hydrogen on Palladium Single-Crystal Surfaces. *Surf Sci* **1974**, *41*, 435-446.

(20) Masel, R. I. *Principles of Adsorption and Reaction on Solid Surfaces*; John Wiley & Sons, Inc., 1996.

(21) Gdowski, G. E.; Felter, T. E.; Stulen, R. H. Effect of Surface-Temperature on the Sorption of Hydrogen by Pd(111). *Surf Sci* **1987**, *181*, L147-L155.

(22) Engel, T.; Kuipers, H. Molecular-Beam Investigation of the Scattering, Adsorption and Absorption of H-2 and D-2 from-on-in Pd(111). *Surf Sci* **1979**, *90*, 162-180.

(23) Andersen, M.; Panosetti, C.; Reuter, K. A Practical Guide to Surface Kinetic Monte Carlo Simulations. *Front Chem* **2019**, *7*.

(24) Kresse, G.; Furthmuller, J. Efficient iterative schemes for ab initio total-energy calculations using a plane-wave basis set. *Phys Rev B* **1996**, *54*, 11169-11186.

(25) Kresse, G.; Furthmuller, J. Efficiency of ab-initio total energy calculations for metals and semiconductors using a plane-wave basis set. *Comp Mater Sci* **1996**, *6*, 15-50.

(26) Kresse, G.; Hafner, J. Ab initio molecular dynamics for liquid metals. *Phys Rev B Condens Matter* **1993**, *47*, 558-561.

(27) Kresse, G.; Hafner, J. Ab initio molecular-dynamics simulation of the liquid-metal-amorphous-semiconductor transition in germanium. *Phys Rev B Condens Matter* **1994**, *49*, 14251-14269.

(28) Perdew, J. P.; Burke, K.; Ernzerhof, M. Generalized gradient approximation made simple. *Phys Rev Lett* **1996**, *77*, 3865-3868.

(29) Perdew, J. P.; Burke, K.; Ernzerhof, M. Generalized gradient approximation made simple (vol 77, pg 3865, 1996). *Phys Rev Lett* **1997**, *78*, 1396-1396.

(30) Monkhorst, H. J.; Pack, J. D. Special Points for Brillouin-Zone Integrations. *Phys Rev B* **1976**, *13*, 5188-5192.

(31) Hammer, B.; Hansen, L. B.; Norskov, J. K. Improved adsorption energetics within density-functional theory using revised Perdew-Burke-Ernzerhof functionals. *Phys Rev B* **1999**, *59*, 7413-7421.
